# Mycorrhizal fungal community structure in tropical humid soils under fallow and cropping conditions

**DOI:** 10.1038/s41598-018-34736-6

**Published:** 2018-11-20

**Authors:** Martin Jemo, Driss Dhiba, Abeer Hashem, Elsayed Fathi Abd_Allah, Abdulaziz A. Alqarawi, Lam-Son Phan Tran

**Affiliations:** 1 Mohammed VI Polytechnic University, Lot 660, Hay Moulay Rachid, 43150 Benguerir, Morocco; 2 OCP-Africa, 2–4, rue Al Abtal, Hay Erraha, 20 200 Casablanca, Morocco; 3 Research & Development, OCP Group, 2-4, rue Al Abtal, Hay Erraha, 20 200 Casablanca, Morocco; 4 Botany and Microbiology Department, College of Science, King Saud University, P.O. Box. 2460, Riyadh, 11451 Saudi Arabia; 5 Mycology and Plant Disease Survey Department, Plant Pathology Research Institute, ARC, Giza, 12511 Egypt; 6 Plant Production Department, College of Food and Agricultural Sciences, King Saud University, P.O. Box. 2460, Riyadh, 11451 Saudi Arabia; 7 Plant Abiotic Stress Research Group & Faculty of Applied Sciences, Ton Duc Thang University, Ho Chi Minh City, Vietnam; 8 Stress Adaptation Research Unit, RIKEN Center for Sustainable Resource Science, 1-7-22 Suehiro-cho, Tsurmi-ku, Yokohama, 230-0045 Japan

## Abstract

Little is known to what extent soil biota, in particular, the mycorrhizae are altered through different fallow durations/types in tropical soils. We found that soil-N, -C, -Al, -K and -Ca contents significantly differed due to the fallow durations/types. Subsequently, the effects of fallow types and soil depths on the diversity, species richness and community structure of arbuscular mycorrhizal (AM) fungi were examined. A higher AM species richness was identified in the cropping than in forest fallow fields, suggesting a positive cropping feedback on the AM community composition. Distribution of the AM species was positively related to soil properties, specifically soil-pH, and soil-Pi, -Ca and -Mg contents. The soil properties conjointly accounted for 78.5% of explained variation in the AM community composition, signifying that the main factors altering the community structure under different fallow and cropping systems were the soil properties. Among the soil chemical characteristics, the soil-pH disclosed a significant explained variation in the AM community composition in the topsoil layer under the short fallow. Structural modeling equation to understand multiple predictive pathways that connect soil properties, fallow practices and AM community structures indicated that soil-C, -N and -Ca contents were highlighted as important factors influencing the AM community compositions.

## Introduction

Shifting cultivation is a predominant and traditional farming practice, alternating cropping sequences and fallow phases in tropical forest, particularly in the humid zone in the south of Cameroon^1,2^. Through fallow periods, the primary natural vegetation and the fertility capital of soils are restored dependently on the associated fallow crops, the length of the fallow period, and the identity of the plant community^2–4^. Therefore, yields of future planted food crops are sustained by fallow process (mainly short-term to medium-term) to meet the food needs of rural smallholdings^5,6^. Increases of crop yields are largely attributed to replenishment of soil nutrient capital via rapid in-depth soil layer mobilization, subsequent crop residue decomposition and release of nutrients to planted crops^2,7,8^. More importantly, the fallows enable the *in situ* build-up of soil biota communities that play key roles in nutrient recycling and suppression of pathogen infections and diseases^9,10^. However, the levels of soil nutrients readily available for uptake by root system are largely dependent on the nature, diversity and composition of plant species used in fallow^11^, length of fallow^12^, soil biota activities in the rhizosphere, and the interactions between the aboveground and belowground ecosystems^13^. It is unfortunate that in many parts of the tropics, the shortening of fallow periods, as a consequence of rapid population growth and increased pressure on agricultural lands, has caused a serious decline in yields of crops traditionally cultivated in mixed-food crop fields. The yield gap is further exacerbated in the actual cultivating systems of the small-scale farms, which can rarely afford organic/inorganic inputs to support crop growth^5,14^. Therefore, soil fertility regeneration through fallow process is no longer feasible; hence, threatening the sustainability of small-scale agriculture^6,12,15^.

From the soil microbiota perspective, microbes play significant roles in the productivity of lands, and are responsible for diverse ecosystem processes and their functioning^16,17^. For example, the great part of nitrogen (N) is recycled via the association between roots of legume plants and rhizobia through the biological atmospheric nitrogen (N_2_) fixation process^18–20^. The soil-turning processes by soil invertebrates contribute to nutrient cycling and accumulation, affecting plant growth^1,21^. The beneficial associations between plant roots and arbuscular mycorrhizal (AM) fungi support uptake and translocation of immobile nutrients, largely N, inorganic phosphate (Pi), carbon (C) and water to host plants in exchange for fixed C source to AM fungi^22,23^. Because of their novel functions in mineral uptake and alleviation of water deprivation-related stresses, the contribution of AM fungi to smallholder agriculture in tropical areas has been recently considered the most important compared with that of other groups of the soil biota^24^. The extent of AM benefits to plants largely depends on their community structure^25–27^, crop species and/or genotype^28^, soil characteristics^29,30^, climatic and geographic context, and the interactions among the above-listed factors^25,27,31^. AM diversity and species richness are in turn largely influenced by the composition of associated plant communities, soil geography, and various agricultural management practices, mainly fertilizer applications, crop rotation, monocropping and tillage practices with selection of soil depths being an important part of tillage practices^32^. However, how the AM composition is reshaped under different soil horizons; this information remains markedly limited, especially under the farming agroecosystem situations in the tropical forest regions, where the cropping activities are almost restricted to the plow layer of soils with direct/indirect consequences on soil chemical and biota changes^33,34^. Furthermore, there has been only scant information about how diverse groups of soil biota are altered by soil depths and their feedback on soil physical-chemical properties^32,35^.

As a result, investigations of AM communities and their importance in tropical ecosystems have been encouraged, especially in the farming systems where the smallholder farmers rarely use fertilizer inputs for their crops^36^. Consequences of the different fallow and cropping conditions imposed by shifting cultivation practices on microbial community and their feedback on plant growth promotion remain largely underinvestigated^5,13^. Increasing evidence depicts ample depletion of nutrients (e.g. soil-Pi and -N), following cropping with inadequate replenishment of the sources^37^. Therefore, the objectives of the present study were (i) to assess the impacts of the fallows, mainly long-term forest and short-term fallows, as well as cropping without fallow on the AM community composition, (ii) to examine how AM community composition was evolved and/or reshaped at different soil depths, and (iii) to assess the effects of soil characteristics, namely soil-pH, and soil-N, -C, -Pi, -Ca, -Mg, -Al and -K contents, and their relevant interactions on the AM community structure, diversity and species richness. Through the knowledge generated, we could gain a better insight into the factors driving the AM community composition and structure in the context of farming systems under the humid tropical soil conditions.

## Methods

### Site description and sampling design

The sampling sites were located in Ngoungoumou (3°18′N, 12°01′E and 609 m above sea level), Metet (3°25′N, 11°45′E and 336 m above sea level) and Nkometou (4°05′N, 11°35′E and 727 m above sea level) in the humid forest of southern Cameroon. The soils at the sampling sites of Ngoungoumou, Metet and Nkometou were classified as *Typic Kandiudox* (Tko), *Typic Kandiudult* (Tku) and *Rhodic Kandiudult* (Rku) subclasses^38^, respectively, according to the USDA soil classification taxonomy (https://www.nrcs.usda.gov/wps/portal/nrcs/main/soils/survey). The long-term mean annual rainfall with bimodal distribution patterns at the sampling sites was 1306, 1380 and 1230 mm for Ngoungoumou, Metet and Nkometou, respectively^39^.

In each of the sampling sites, cropping fields and two kinds of fallow fields were selected, with four replicates each being sampled in two depths (0–10 and 10–20 cm) for assessment of the AM community composition and structure. The two fallow fields were the long-term forest fallow field of 20-year-old and the short-term fallow field dominated by *Chromoleana odorata*, an invasive weed species of 4- to 6-year-old. The cropping fields were dominated with planted food crops, mainly groundnut (*Arachis hypogaea*) and cassava (*Manihot esculenta*) grown in association with cocoyam (*Colocasia esculenta*), banana (*Musa* sp.), maize (*Zea mays*), yam (*Discorea rotundata*) and a number of leafy vegetables (*Amaranthus cruentus*, *Corchorus olitorius*, *Solanum scabrum* and *Vernonia calvoana*). The farmers traditionally managed their fields from land preparation, planting/sowing, weeding to harvesting. The soil sampling activities were conducted during vegetative growth stage of food crops, which corresponded to approximately 48–56 days after planting/sowing.

To carry out the sampling of soils from fields, sixteen core soil samples were collected from 0–10 and 10–20 cm depths, respectively, in four delimited 10 m × 10 m plots at each site. All core samples were bulked and transferred to the laboratory. In total, 72 composite soil samples were collected from all three sites and stored at 4 °C for subsequent analyses. As the first step, the soils were sieved through a 4-mm mesh, and then divided into two pools. The first pool was used to establish the bioassay culture, and the second pool was sieved to pass a 1-mm mesh and was then used for chemical analyses (e.g. measurements of soil-pH, as well as contents of total soil-C and -N, and available soil-Pi and other elements).

### Chemical analyses

After the soil samples were shaken in water for 16 h, soil-pH was determined in the soil suspension (1:2.5, v:v) with the help of a Corning 125 pH meter (Corning, Life Sciences, Amsterdam, Netherlands). Four mg of each sample collected from the respective sites were pulverized in a ball mill (MM200, Retsch, Germany). The total soil-N and -C contents in prepared, dried samples were measured by using an elemental analyser (Flash EA 1112, Thermo Electron, Ecublens, Switzerland). For determination of the soil-Ca, -Mg, -Al and -K contents, 500 mg of soil were incinerated in a muffle furnace at 550 °C for 8 h. Subsequently, the ashes were dissolved in 2 mL of concentrated HNO_3_ at 180 °C and were then made up to 50 mL with ultrapure water. The soil-Ca, -Mg, -Al and -K contents were measured in the 20-fold diluted extracts using an inductively coupled plasma mass spectrometry (ICP-MS, Agilent 7500c, Agilent, Basel, Switzerland). To determine the inorganic Pi content in soil, the Bray-I method was used. Briefly, 30 mL of Bray-I extractant were added to 3 g of air-dried soil sample. The mixture (1:10, soil:solution ratio) was shaken for 5 min and filtered, and the soil-Pi content was measured by colorimetric change^40^.

### Bioassay

The experiment was designed in a factorial arrangement with the following factors tested: (i) factor 1, fallow types (F) with three levels: long-term forest fallow, short fallow and cropping fields; (ii) factor 2, soil origins (S) with three levels: Tko, Tku and Rku soils; and (iii) factor 3, sampling depths (D) with two levels: 0–10 and 10–20 cm. To establish the bioassay, 200 g fresh soil of each sample were mixed together with 800 g of autoclaved sand–quartz substrate (grain size between 0.7 and 1.2 mm), and 1 L capacity of the substrate mixture was used to fill up each pot. Subsequently, one-week-old pre-germinated seedlings of leek (*Allium porrum*) and red clover (*Trifolium pratense*) were manually transplanted to the pots. The leek and red clover plants were grown for 6 months in growth chamber PGC20 (Conviron, Winnipeg, Canada), and then they were replaced by pre-germinated alfalfa (Medicago sativa) and ryegrass (Lolium multiflorum) seedlings that were subsequently grown for 6 months in a growth chamber. The following growth chamber conditions were maintained before the soils were sampled for AM microscopic observations and molecular analyses: 16/8 h day/night time regime with a photon flux density of 231 µmol s^−1^ m^−2^ during the daytime, 28/18 °C day/night temperature and 75–85% relative air humidity. Pots were automatically watered with deionized water using a tensiometer (Tropf-Blumat, Weninger GmbH, Telfs, Austria) to maintain 60% water holding capacity. The pots were fertilized once a week with 25 mL of 8-fold-concentrated Hoagland nutrient solution^41^.

### Microscopic observations

Spore extraction of the AM species was carried out using the modified wet sieving method followed by sucrose-gradient centrifugation as previously described^42^. Briefly, 25 g of wet substrate mixture were washed thoroughly with tap water through a 500-μm mesh, and then the suspension was collected using a 40-μm mesh sieve and transferred into a 50-mL centrifuge tube. Ten mL of 2.5 M sucrose solution were added followed by a centrifugation at 2000 × g for 2 min. After centrifugation, the supernatant was poured into a 40-μm mesh sieve and quickly rinsed with tap water. Turgid (viable) individuals were counted and grouped according to the species morphology. Permanent slides were prepared for each species using either polyvinyl alcohol alone or mixed with Melzer’s solution (1:1), and were then observed under the Olympus AX70 microscope (Olympus Optical, Tokyo, Japan). Different AM species recorded were compared with the recent nomenclature of AM fungi as previously described^43^. Soil moisture content was determined after a subsample of fresh soil from each pot was oven-dried at 105 °C for 48 h, and the dry weight was recorded. Spore density, expressed as the number of AM spores per 25 g of fresh substrate, was adjusted to the dry matter of soil using the soil moisture content values. The Shannon-Weaver diversity index (H) was calculated for different F, S and D using the following equation Eq. 1:

1 $${\rm{H}}=-\,\sum _{i=1}^{N}pi\,\times \,\mathrm{ln}\,(pi)$$

where *p*i was the spore abundance of the i^th^ species among all *N* species identified in a sampling depth of a soil origin under a respective fallow or cropping field.

The species abundance distribution (SAD) of each identified AM species^44,45^ was determined based on the equation Eq. 2:

2 $$\begin{array}{c}{\rm{SAD}}=({\rm{Total}}\,{\rm{spore}}\,{\rm{number}}\,{\rm{of}}\,{\rm{each}}\,{\rm{AM}}\,{\rm{species}}\times 100)\\ \,\,\,/({\rm{Total}}\,{\rm{number}}\,{\rm{of}}\,{\rm{all}}\,{\rm{identified}}\,{\rm{AM}}\,{\rm{species}})\end{array}$$

The AM species richness, which indicates the number of the AM species found in soil, was determined as previously described^46^.

### Molecular analysis

To complement the morphological identification, sequencing of the 28S ribosomal DNA (rDNA) of the respective AM isolates observed under the microscope was conducted according to a previously published method^42^. Briefly, DNA was recovered from fresh and juvenile spores of each AM species, and amplified by the polymerase chain reaction (PCR) using the LR1 (5′-GCATATCAATAAGCGGAGGA-3′) and LR2 (5′-GTCGTTTAAAGCCATTACGTC-3′) primers^47^. The PCR reactions were performed in an automated thermal cycler (Biometra T-Gradient, Leusden, Netherlands) with an initial denaturation step at 94 °C for 3 min, amplification with 36 cycles (denaturation for 15 s at 94 °C, annealing for 90 s at 50 °C, elongation for 90 s at 72 °C), and final elongation for 10 min at 72 °C. The PCR products were visualized on 0.8% agarose gel with ethidium bromide under UV light to confirm their integrity. The PCR products were then purified using a gel extraction kit (Qiagen AG, Hombrechtikon, Switzerland), following the manufacturer’s procedure, and cloned into the pGEM-T plasmid (Promega, Dübendorf, Switzerland). Sequencing of the cloned PCR products was carried out using a Sanger DNA sequencer (Microsynth, Balgach, Switzerland), and each sequence was manually edited and blasted against GenBank for homology search using the BLASTN algorithm. Multiple sequence alignment was conducted using BioEdit (v.7.2.1)^48^. Phylogenetic analysis was performed by the Neighbour-joining method with a bootstrap value of 1000 using the MEGA5 software^49^. We chose the AM species from the GenBank to represent as broadly as possible the phylogenetic diversity within the phylum Glomeromycota and using *Morchella esculenta* and *Fusarium sambucinum* as outgroup species.

### Data analysis

A three-way analysis of variance (ANOVA) was performed using the General Linear Procedure ‘Proc GLM’ in the Statistical Analysis System (SAS) software (v. 9.2)^50^ to test the effects of F, S, D, and their interactions on the soil parameters and the AM community structure and diversity. On the basis of the significant Fisher’s (*f*) values, a Least-Means test (Least-Means/PDIFF option) was performed to separate means among the F, S and D. This was carried out exclusively when the Student’s *t*-test from the ANOVA resulted in a significant *p*-value (*p* < 0.05). Levels of significance are given by ‘*ns*’ (not significant, *p* > 0.05), *p < 0.05, **p < 0.01 and ***p < 0.001.

A principal component analysis (PCA) was conducted to explore the interrelated effects of soil characteristic data (e.g. soil-pH, and soil-N, -C, -Pi, -Al, -K, -Ca and -Mg contents) obtained in the two depths of the forest, short fallow and cropping fields. A redundancy analysis (RDA)-based variation-partitioning method was used to extract the effects of explanatory variables (i.e. soil-pH, and soil-N, -C, -Pi, -Al, -K, -Ca and -Mg contents), and factorial predictors (i.e: F, S and D) on the AM community composition using the ‘rda’ function in the ‘vegan’ library of the R program. Prior to RDA, a detrended correspondence analysis (DCA) was used to extract the axes of maximum variation in the AM species composition, and to identify the gradient lengths from original data. Biplots of the DCA and RDA were displayed using the CANOCO software (v.5.2)^51^. The response curves of individual AM species against the DCA axis 1 (DCA1) were performed, and the model fitted for the respective AM species was presented. The significant levels for both canonical axes were determined, and their *f*-values were reported following the Monte Carlo permutation test with 499 permutations^52^. Furthermore, the correlations between individual soil characteristics (SC) and different AM species were analyzed by generalized additive model using the Gaussian distribution and log (x + 1)-transformed SAD values. When the effects of explanatory variables were significantly correlated with the AM species composition, a variation-partitioning analysis was performed adopting the approach previously described^53^. A prior stepwise selection of explanatory variables in each of the two groups of predictors (quantitative and factor estimators) was carried out with candidate predictor inclusion based on the significant values^54^. Venn diagram of the variation-partitioning results was then created. The adjusted *p*-values for multiple comparison tests reporting statistically significant influences among the analyzed soil properties (i.e. soil-pH, and soil-N, -C, -Pi, -Al, -K, -Ca and -Mg contents) on the AM species composition were determined based on the Bonferroni correction test.

The structural equation modeling (SEM) was used to estimate the multiple cause-effect relationships between the fallow practices, SC and AM composition^32^. The SEM model allows the specification of multiple predictive pathways among model variables to account for their influence on each other based on the two sub-model approach: the structural model and measurement model. The structural model was built from the analyzed soil parameters, results of the RDA-based variation-partitioning and ANOVA analyses, taking into account the published literature on the AM studies. An explanatory stepwise procedure was conducted through the use of modification indices to increase the fit of the models by relaxing imposed constraints from the measured vairables. The final models were reached after their validation using the maximum criteria required for the chi-square (χ^2^), the standardized root mean square residual (SRMR), and the comparative fit index (CFI) based on the maximum-likelihood analysis. The path coefficients indicated the degree (strong/weak) of relationship, while the thick and thin arrows indicated direct or indirect effects from the measured variables that were well fitted into the models.

## Results

### Variations in soil properties across fallow types, soil origins and sampling depths

Results from the one-way ANOVA showed significant different effects due to the fallow types (F) on six of the eight examined soil chemical properties, with the exception of the soil-Mg and -Pi contents (Table 1). The soil-pH values ranged from 4.3 to 7.2, and were acidic regarding the Tko and Tku, and were neutral with reference to the Rku soils (Table 2). Furthermore, the soil-pH exhibited higher values in the 0–10 cm depth of the Tko soil under the cropping and short fallow as compared with that of the forest fallow. Both the cropping and short fallow soils displayed greater soil-pH values relative to that of the forest fallow in both top and lower layers (0–10 and 10–20 cm depths) of the Tku soil. We also found that the cropping displayed the highest soil-pH value in comparison with the forest and short fallows in the 0–10 cm sampling layer of the Rku soil. In addition, the cropping fields recorded greater soil-C, -N and -Al contents than the forest and short fallows in both the top and subsoil layers of the Tko soil. We also noticed that the cropping soils displayed higher soil-C, -N, -Pi, -Mg, -Al and -Ca contents than the forest and short fallows in both two sampling depths of the Rku soil. Additionally, the cropping and short fallow exhibited greater soil-C contents than the forrest fallow in both sampling depths of the Rku soil. With respect to the Tku, the cropping fields showed an increasing tendency in soil-Mg and -Al contents as compared with the other two fallows in the 0–10 cm soil layer. We also found that the cropping and short fallow fields displayed the lowest soil-N contents as compared with that of the forest fallow fields in the 10–20 cm depth of Tku soil (Table 2).

**Table 1:** Results of the three-way ANOVA testing the differential effects of fallow types (F, three levels; forest fallow, short fallow and cropping conditions), soil origins (S, three levels; *Typic Kandiudox*, *Typic Kandiudult* and *Rhodic Kandiudult*), sampling depths (D, two levels; 0–10 cm and 10–20 cm), and their interactions on the soil chemical properties.

	Fallow types (F)	Soil origins (S)	Sampling depths (D)	F × S	F × D	S × D	F × S × D
Soil-pH	24.6 (***)	357.1 (***)	64.8 (***)	4.4 (**)	0.2 (*ns*)	7.4 (**)	3.4 (*)
Soil-N content (mg kg^−1^)	3.8 (**)	6.5 (**)	101.8 (***)	2.1 (*ns*)	3.1 (*)	1.1 (*ns*)	0.6 (*ns*)
Soil-C content (g kg^−1^)	5.8 (**)	7.8 (**)	94.8 (***)	1.3 (*ns*)	4.6 (*)	2.7 (*ns*)	0.6 (*ns*)
Soil-Mg content [cmol (+) kg^−1^]	2.0 (*ns*)	4.5 (*)	44.5 (***)	1.2 (*ns*)	1.3 (*ns*)	7.4 (**)	1.5 (*ns*)
Soil-Al content [cmol (+) kg^−1^]	12.9 (***)	2.1 (*ns*)	1.0 (*ns*)	1.4 (*ns*)	2.5 (*ns*)	5.0 (*)	0.9 (*ns*)
Soil-K content [cmol (+) kg^−1^]	5.2 (**)	7.2 (**)	3.0 (*ns*)	1.8 (*ns*)	1.4 (*ns*)	6.3 (**)	4.2 (**)
Soil-Ca content [cmol (+) kg^−1^]	14.0 (***)	41.2 (***)	68 (***)	6.0 (***)	2.5 (*ns*)	7.0 (**)	3.8 (**)
Soil-Pi content (mg kg^−1^)	1.2 (*ns*)	6.2 (**)	35.6 (***)	8.5 (***)	5.6 (**)	25.4 (***)	3.6 (*)

*f*-values (*n* = 4 replicates) are shown. Levels of statistically significant differences are given by ‘*ns*’ (not significant, *p* ≥ *0*.*05*); *p < 0.05; **p < 0.01; ***p < 0.001. N, nitrogen; C, carbon; Mg, magnesium; Al, aluminum; K, potassium; Ca, calcium; Pi, phosphate.

**Table 2:** Chemical properties of soils under different fallow types, soil origins and sampling depths before the bioassay establishment.

	Tko		Tku		Rku	
	0–10 cm	10–20 cm	0–100–10 cm	10–20 cm	0–10 cm	10–20 cm
**Soil-pH**						
Forest fallow	4.8 ± 0.15 bC	4.6 ± 0.19 aC	4.4 ± 0.10 cD	4.3 ± 0.07 bD	6.6 ± 0.05 cA	6.3 ± 0.15 aB
Short fallow	5.4 ± 0.09 aD	4.7 ± 0.15 aE	5.7 ± 0.14 aC	4.7 ± 0.05 aE	6.7 ± 0.11 bA	6.4 ± 0.03 aB
Cropping	5.5 ± 0.20 aC	4.8 ± 0.04 aD	5.2 ± 0.11 bC	4.8 ± 0.11 aD	7.2 ± 0.20 aA	6.5 ± 0.17 aB
**Soil-C content [g kg** ^**−1**^ **]**						
Forest fallow	23.1 ± 4.4 bA	16.1 ± 2.7 aB	22.6 ± 3.7 aA	12.8 ± 0.33 aC	22.4 ± 1.9 cA	16.6 ± 1.5 bB
Short fallow	33.6 ± 6.0 aA	13.1 ± 2.3 aC	25.8 ± 2.2 aA	12.0 ± 0.62 aC	29.0 ± 1.3 bA	19.7 ± 1.2 aB
Cropping	40.1 ± 5.4 aA	16.6 ± 2.8 aC	25.9 ± 1.9 aB	10.2 ± 0.22 bD	33.4 ± 2.9 aA	20.7 ± 1.4 aC
**Soil-N content [g kg** ^**−1**^ **]**						
Forest fallow	1.9 ± 0.38 cA	1.2 ± 0.19 aB	1.8 ± 0.26 aA	1.1 ± 0.02 aB	1.8 ± 0.18 bA	1.1 ± 0.11 bB
Short fallow	2.3 ± 0.39 bA	1.1 ± 0.20 aBC	1.9 ± 0.12 aA	0.9 ± 0.03 bC	2.0 ± 0.07 bA	1.3 ± 0.09 aB
Cropping	3.0 ± 0.33 aA	1.3 ± 0.27 aD	1.8 ± 0.10 aC	0.8 ± 0.04 cE	2.6 ± 0.26 aB	1.3 ± 0.09 aD
**Soil-Pi content [mg kg** ^**−1**^ **]**						
Forest fallow	5.1 ± 0.66 aA	3.1 ± 0.91 aB	3.6 ± 0.68 aB	1.3 ± 0.29 aC	1.2 ± 0.30 bC	4.1 ± 0.12 aAB
Short fallow	4.2 ± 0.61 abA	1.5 ± 0.15 bC	4.4 ± 0.34 aA	1.6 ± 0.14 aC	3.9 ± 0.65 aA	2.8 ± 0.15 bB
Cropping	3.6 ± 0.49 bB	1.9 ± 0.20 bC	3.9 ± 0.31 aB	1.5 ± 0.08 aC	4.5 ± 0.48 aAB	5.2 ± 0.34 aA
**Soil-Mg content [cmol** (+) **kg**^**−1**^**]**						
Forest fallow	1.2 ± 0.29 aA	0.8 ± 0.13 bB	0.9 ± 0.16 bB	0.8 ± 0.06 abB	1.2 ± 0.03 bA	0.5 ± 0.08 cC
Short fallow	1.2 ± 0.31 aB	0.8 ± 0.08 bC	1.1 ± 0.11 abB	0.7 ± 0.08 bC	1.8 ± 0.14 aA	0.7 ± 0.07 bC
Cropping	1.0 ± 0.18 aB	1.1 ± 0.14 aB	1.2 ± 0.07 aB	0.9 ± 0.08 aC	2.0 ± 0.13 aA	0.9 ± 0.11 aBC
**Soil-Al content [cmol** (+) **kg**^**−1**^**]**						
Forest fallow	34.7 ± 6.8 bAB	42.4 ± 2.0 bA	34.3 ± 1.2 bB	42.5 ± 1.5 aA	41.0 ± 3.7 cA	34.8 ± 0.83 bB
Short fallow	39.9 ± 7.6 abAB	42.8 ± 3.6 abAB	38.0 ± 2.7 bB	34.1 ± 2.6 bBC	47.1 ± 1.4 bA	32.2 ± 1.5 bC
Cropping	45.0 ± 2.9 aB	46.3 ± 0.98 aB	46.7 ± 2.8 aAB	40.6 ± 3.3 aB	54.7 ± 4.2 aA	50.6 ± 2.8 aA
**Soil-K content [cmol** (+) **kg**^**−1**^**]**						
Forest fallow	0.5 ± 0.05 aB	0.4 ± 0.03 bC	0.5 ± 0.03 aB	0.9 ± 0.09 aA	0.6 ± 0.13 aB	0.6 ± 0.08 aB
Short fallow	0.4 ± 0.09 bBC	0.4 ± 0.02 bB	0.4 ± 0.02 bB	0.7 ± 0.07 bA	0.5 ± 0.08 bB	0.3 ± 0.04 bC
Cropping	0.4 ± 0.03 bC	0.5 ± 0.07 aBC	0.5 ± 0.04 aAB	0.5 ± 0.05 cAB	0.5 ± 0.08 bABC	0.6 ± 0.09 aAB
**Soil-Ca content [cmol** (+) **kg**^**−1**^**]**						
Forest fallow	3.7 ± 0.92 aB	2.5 ± 0.65 abB	3.7 ± 0.79 abB	1.2 ± 0.18 bC	6.1 ± 0.34 cA	3.1 ± 1.16 bB
Short fallow	6.6 ± 2.03 aB	1.9 ± 0.34 bC	4.7 ± 0.63 aB	1.3 ± 0.16 bD	9.5 ± 0.79 bA	4.6 ± 0.31 bB
Cropping	5.5 ± 1.3 aB	3.5 ± 0.62 aBC	3.6 ± 0.38 bC	2.6 ± 0.28 aD	16.0 ± 1.9 aA	6.0 ± 0.96 aB

Four replicates (*n* = 4) per site were used. C, carbon; N, nitrogen; Pi, phosphate; Mg, magnesium; Al, aluminum; K, potassium; Ca, calcium. Numbers followed by different small letters within a column are significantly different (*p* < 0.05) in comparing the fallow types (forest fallow, short fallow and cropping conditions) within a sampling depth of each soil origin. Numbers followed by different capital letters within a row are significantly different (*p* < 0.05) in comparing the two soil depths (0–10 cm and 10–20 cm) in all three soil origins (Tko, *Typic Kandiudox*; Tku, *Typic Kandiudult*; Rku, *Rhodic Kandiudult*).

With regard to the sampling depths (D), the ANOVA exhibited significant *f-*values for the majority of analyzed soil characteristics (SC), except the soil-Al and -K contents (Table 1); with the 0–10 cm sampling depth displaying the highest values of the soil-pH, and soil-C, -N and -Ca contents, irrespective of the F and soil origins (S) (Table 2). Furthermore, the Rku soil generally displayed greater values of soil-pH than the Tko and Tku soils in both soil depths (Table 2). Among the interactions tested, the F × S interaction resulted in significantly different effects on the soil-pH, and soil-Ca and -Pi contents (Table 1). Similarly, the S × D interaction displayed statistically significant *f*-values with respect to the soil-pH, and soil-Mg, -Al, -K, -Ca and -Pi contents, while the F × D interaction showed significantly different influences on the soil-N, -C and -Pi levels (Table 1). Finally, the F × S × D interaction exhibited significantly different effects on the soil-pH, and soil-K, -Ca and -Pi contents following a 3-way ANOVA (Table 1).

**Figure 1:**
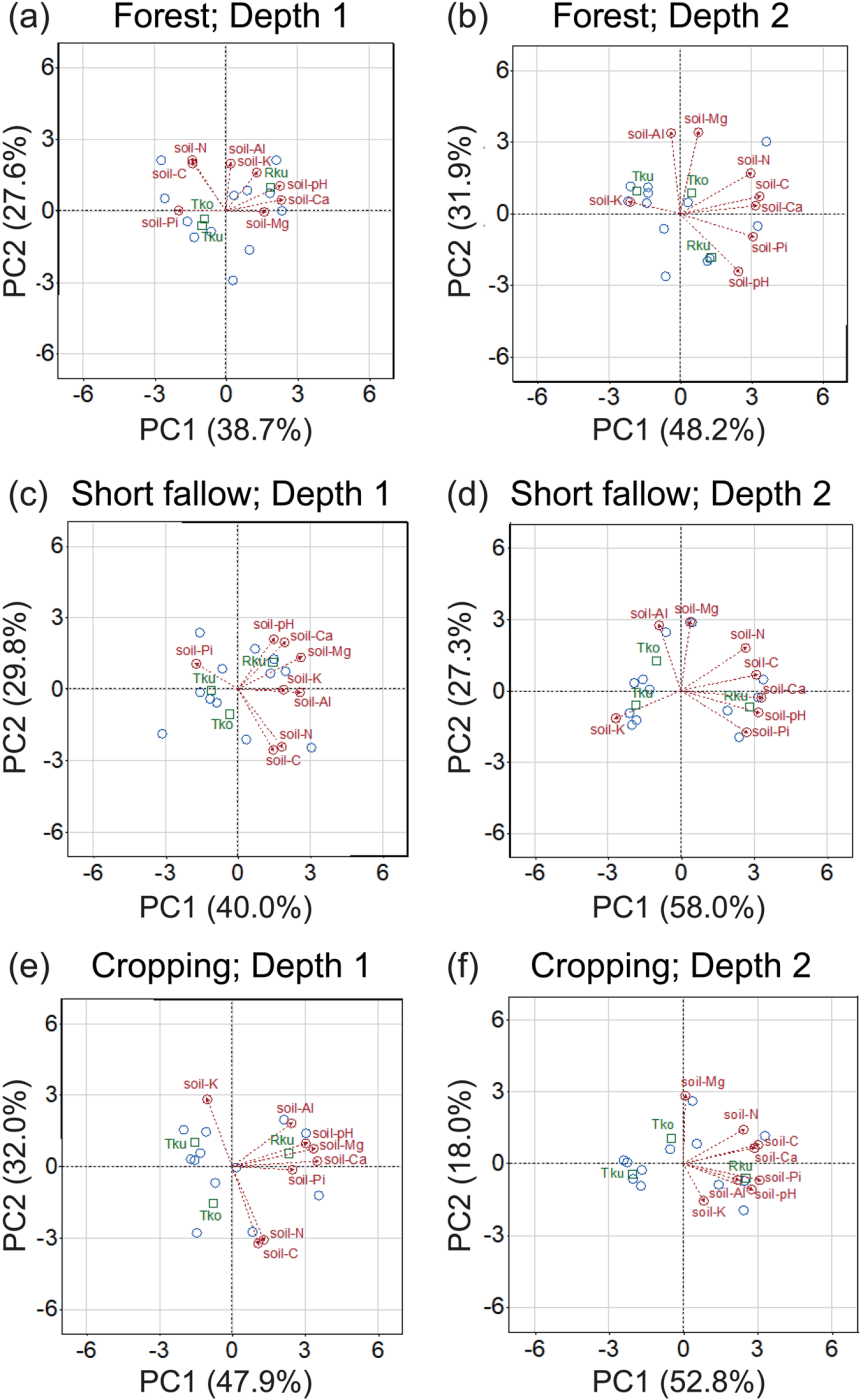
Biplots of the principal component (PC) analysis of soil characteristics by fallow types (forest fallow, short fallow and cropping conditions) and sampling depths (0–10 cm and 10–20 cm). (**a**,**b**) Forest fallow, (**c**,**d**) short fallow, and (**e**,**f**) cropping fields with the two sampling depths (Depth 1, 0–10 cm; Depth 2, 10–20 cm). Tko, *Typic Kandiudox*; Tku, *Typic Kandiudult*; Rku, *Rhodic Kandiudult*; soil-pH, pH measured in soil suspension; soil-N, nitrogen content; soil-C, carbon content; soil-Mg, magnesium content; soil-Al, aluminium content; soil-K, potassium content; soil-Ca, calcium content; soil-Pi, inorganic phosphate content in soil.

The PCA investigating how the SC were interrelated under the different F and D allowed us to identify two major components accounting for the total variance explained (TVE) with cumulative values ranging from 66.3 to 85.3% of TVE (Fig. 1; Supplementary Table 1). PC1, the most informative component, displayed the highest, while PC2 exhibited the lowest TVE values in both the top and sublayer soils under the different F conditions (Supplementary Table 1). The loading matrix correlating original variables from the PCs indicated noticeable and positive loadings from the soil-Ca content for the PC1 in both 0–10 and 10–20 cm soil depths of all the F (Fig. 1a–f; Supplementary Table 1). Additionally, the soil-N and -C variables consistently displayed positive, high loading into the PC1 direction in the 10–20 cm soil depth irrespective of the F (Fig. 1b,d,f; Supplementary Table 1). With respect to soil-Pi variable, high and positive loadings were disclosed for the PC1 in the 10–20 cm soil, irrespective of the F, while moderate and negative loadings were observed for the PC1 in the 0–10 cm soil depth of the forest and short fallow conditions (Fig. 1a–f; Supplementary Table 1). The soil-K content revealed a moderate and negative loading value into the PC1 in the 10–20 cm soil layer under the forest fallow, while a moderate and positive loading was recorded in the 0–10 cm soil depth under the short fallow conditions (Fig. 1b,c; Supplementary Table 1). Finally, we noticed positive and high loading scores for PC1 from the soil-Mg variable in the 0–10 cm soil depth under the short fallow and cropping conditions (Fig. 1c,e; Supplementary Table 1).

As for the PC2, the soil-N and -C variables exhibited high and positive loadings in the 0–10 cm soil depth of the forrest fallow, while high and negative loadings in the 0–10 cm soil layer under the short fallow and cropping conditions (Fig. 1a,c,e; Supplementary Table 1). The loadings from the soil-Al variable into the direction of PC2 indicated high and positive scores in the 0–10 cm depth of the forest fallow and in the 10–20 cm depth of the forest and short fallows (Fig. 1a,b,d; Supplementary Table 1). In addition, the soil-Mg content displayed positive and high loading values in the 10–20 cm soil depth of all examined forrest fallow, short fallow and cropping practices in the direction to the PC2 (Fig. 1b,d,f; Supplementary Table 1).

### Identification and classification of the AM species in soils after the fallows and cropping

The 28S rDNA fragments were amplified from the spores of different AM species, which were detected under a microscope, and were sequenced. Subsequently, the sequence data (accession numbers provided in Supplementary Fig. 1) were blasted with available sequences in GenBank for homology search using the BLASTN. A phylogenic tree was then constructed using the sequence data of the 28S rDNA fragments to determine the phylogenic relationship of the individually identified AM species (Supplementary Fig. 1). Up to 12 different AM species were detected from various fallow types, soil origins and sampling depths. Using the nomenclature of AM fungi previously reported^43^, the identified AM fungi could be classified into the Glomeraceae, Diversporaceae, Acaulosporaceae, Archeosporaceae, Gigasporaceae, Ambisporaceae and Paraglomaraceae families (Supplementary Fig. 1).

### Impact of fallow types, soil origins and sampling depths on the AM species abundance distribution, richness, diversity and number of their spores

Results of the 3-way ANOVA testing the effects of fallow types (F), soil origins (S) and sampling depths (D) on the species abundance distribution (SAD) of the AM fungi are presented in Table 3. Significantly different effects of the F were detected on the SAD of four out of the 12 recorded AM species, namely *Acaulospora scrobiculata*, *A*. *mellea*, *Rhizophagus intraradices* and *Ambispora* sp., while the effects of S on the SAD of AM species displayed significant *f*-values for *A*. *mellea*, *Cetraspora pellucida*, *Racocetra tropicana* and *Glomus microaggregatum* and *Ambispora* sp. (Table 3). Significantly different effects of the D were also observed on the SAD of five out of 12 detected AM species, including *A*. *scrobiculata*, *A*. *mellea*, *Paraglomus occultum*, *Gigaspora decipiens* and *R*. *intraradices*. Concerning their interaction effects, the F × S interaction revealed significant *f*-values for *A*. *mellea* and *P*. *occultum*, whereas F × D and S × D interactions showed significantly different effects on *A*. *scrobiculata*, *C*. *pellucida* and *R*. *tropicana*, and *A*. *scrobiculata*, *A*. *mellea* and *A*. *paulineae*, respectively. Additionally, the triple F × S × D interaction exhibited critical influence on SAD of six AM communities, namely *A*. *scrobiculata*, *A*. *mellea*, *C*. *pellucida*, *R*. *tropicana*, *A*. *gerdemannii* and *Ambispora* sp. (Table 3).

**Table 3:** Results of the three-way ANOVA testing the differential effects of fallow types (F, three levels; forest fallow, short fallow and cropping conditions), soil origins (S, three levels; *Typic Kandiudox*, *Typic Kandiudult* and *Rhodic Kandiudult*), sampling depths (D, two levels; 0–10 cm and 10–20 cm), and their interactions on the arbuscular mycorrhizal species abundance distribution, species richness, Shannon-Weaver (S-W) diversity index and spore number.

	Fallow types (F)	Soil origins (S)	Sampling depths (D)	F × S	F × D	S × D	F × S × D
**Species abundance distribution**							
*Acaulospora scrobiculata*	5.7 (**)	2.8 (*ns*)	11.1 (**)	0.4 (*ns*)	3.4 (*)	9.0 (***)	5.1 (**)
*Acaulospora mellea*	4.1 (*)	34.0 (***)	26.3 (***)	18.5 (***)	1.2 (*ns*)	11.5 (***)	5.2 (**)
*Acaulospora paulineae*	0.9 (*ns*)	0.05 (*ns*)	0.1 (*ns*)	0.5 (*ns*)	2.3 (*ns*)	15.9 (***)	0.9 (*ns*)
*Paraglomus occultum*	1.5 (*ns*)	1.4 (*ns*)	5.6 (*)	3.8 (*)	1.5 (*ns*)	1.4 (*ns*)	1.7 (*ns*)
*Cetraspora pellucida*	2.5 (*ns*)	3.4 (*)	1.2 (*ns*)	2.3 (*ns*)	3.3 (*)	2.2 (*ns*)	3.1 (*)
*Racocetra tropicana*	2.5 (*ns*)	3.5 (*)	2.4 (*ns*)	2.4 (*ns*)	3.5 (*)	2.3 (*ns*)	3.2 (*)
*Gigaspora decipiens*	1.4 (*ns*)	2.4 (*ns*)	5.6 (*)	0.9 (*ns*)	2.5 (*ns*)	0.9 (*ns*)	1.2 (*ns*)
*Gigaspora margarita*	1.0 (*ns*)	1.2 (*ns*)	1.0 (*ns*)	1.0 (*ns*)	1.0 (*ns*)	1.0 (*ns*)	1.0 (*ns*)
*Glomus microaggregatum*	0.4 (*ns*)	7.6 (**)	0.1 (*ns*)	2.2 (*ns*)	0.6 (*ns*)	0.3 (*ns*)	1.8 (*ns*)
*Rhizophagus intraradices*	4.9 (*)	2.0 (*ns*)	10.0 (**)	1.4 (*ns*)	1.4 (*ns*)	2.2 (*ns*)	2.0 (*ns*)
*Ambispora gerdemannii*	2.1 (*ns*)	1.5 (*ns*)	1.6 (*ns*)	1.0 (*ns*)	1.4 (*ns*)	1.0 (*ns*)	2.9 (*)
*Ambispora* sp.	4.5 (*)	24.8 (***)	3.7 (*ns*)	2.2 (*ns*)	1.4 (*ns*)	2.2 (*ns*)	5.5 (**)
Species richness	11.3 (***)	21.1 (***)	13.3 (***)	4.7 (**)	2.3 (*ns*)	18.0 (***)	5.7 (**)
S-W diversity index	3.6 (*)	14.9 (***)	12.6 (***)	1.3 (*ns*)	0.3 (*ns*)	6.7 (**)	4.3 (**)
Spore number (g^−1^ soil)	5.2 (**)	0.7 (ns)	11.2 (**)	1.1 (*ns*)	1.5 (*ns*)	2.3 (*ns*)	1.9 (*ns*)

Data from the microscopic observations were log-transformed before the analysis. *f*-values (*n* = 4 replicates) are shown, and asterisks indicate statistically significant differences. *p* < 0.05; **p < 0.01; ***p < 0.001; *ns*, not significant (*p* ≥ 0.05).

**Figure 2:**
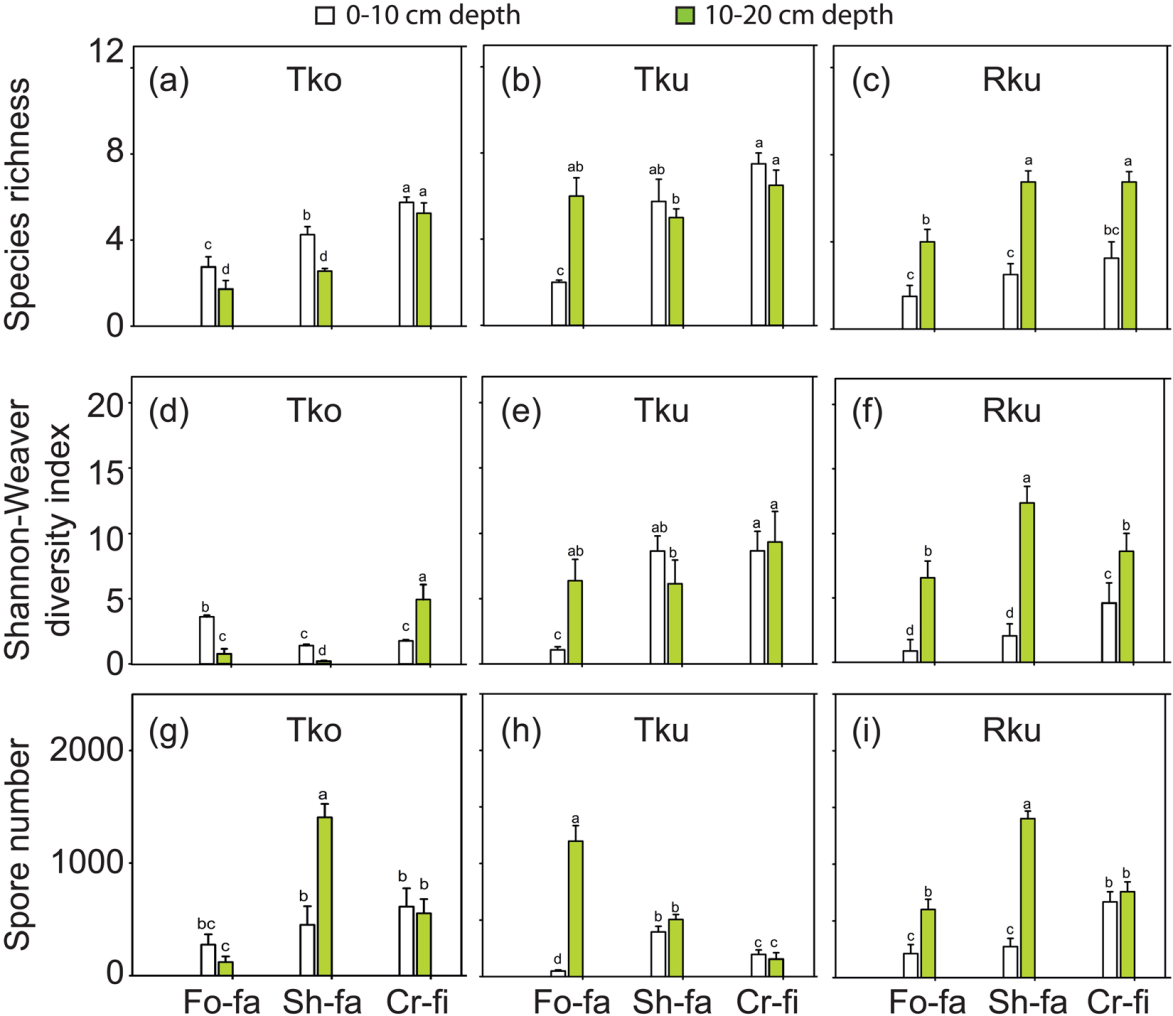
Species richness (**a**–**c**), Shannon-Weaver diversity index (**d**–**f**) and arbuscular mycorrhizal spore number (g^-1^ soil) (**g**–**i**) in two sampling depths (0–10 cm and 10–20 cm) of three soil types (Tko, *Typic Kandiudox*; Tku, *Typic Kandiudult*; Rku, *Rhodic Kandiudult*) under the forest fallow, short fallow and cropping conditions. Mean values and standard errors are shown (*n* = 4). Different letters indicate significant differences between the two soil depths for all the fallow types in each soil origin according to the Fisher’s test following a one-way ANOVA (*p* < 0.05). Fo-fa, forest fallow; Sh-fa, short fallow; Cr-fi, cropping field.

The 3-way ANOVA also demonstrated significantly different effects of the F on the AM species richness (Table 3). The cropping fields generally recorded greater AM species richness (α-diversity) as compared with the forest fallow in both two sampling depths of the Tko and Rku soils, according to a one-way ANOVA (Fig. 2a–c). Furthermore, the S also displayed significantly different influences on the AM species richness (Table 3). Moreover, the Fisher’s test indicated that the effects of the D on the AM species richness were also highly significant (Table 3), with the 10–20 cm depth of the Rku soil exhibiting greater values of AM species richness than the 0–10 cm depth under all fallow and cropping conditions (Fig. 2c). Overall, the AM species richness ranged from 1.5 to 7.5; with the 0–10 cm sampling depth of the Rku under the forest fallow displaying the lowest, and the 0–10 cm sampling depth of the cropping field of Tku soil disclosing the highest value of the AM species richness (Fig. 2a–c). Among the tested interactions, F × S, S × D and F × S × D interactions showed significantly different influences on the AM species richness (Table 3).

With respect to the AM species diversity, the Shannon-Weaver diversity index ranged from 0.2 to 12.3 (Fig. 2d–f). The 10–20 cm depth of Tko soil displayed the lowest, and the same sampling depth of the Rku soil disclosed the highest Shannon-Weaver diversity value under the short fallow (Fig. 2d,f). The 3-way ANOVA testing the effect of the F on the Shannon-Weaver diversity index noted a significant *f*-value (Table 3). For instance; within the Tko soil, the forest fallow and cropping fields showed the highest diversity index in 0–10 and 10–20 cm sampling depth, respectively (Fig. 2d). The 3-way ANOVA also revealed significantly different effects of the S on the Shannon-Weaver diversity index (Table 3). In addition, the effects of D on the Shannon-Weaver diversity index were greatly and significantly different according to the 3-way ANOVA (Table 3), displaying remarkable differences between the 0–10 and the 10–20 cm depths (Fig. 2d–f). For instance, the 10–20 cm depth of the Rku soil recorded higher Shannon-Weaver diversity values than the 0–10 cm depth of the same soil type in all forest fallow, short fallow and cropping fields (Fig. 2f). Two out of the four tested interactions, namely the S × D and F × S × D interactions were significantly different in terms of the Shannon-Weaver diversity index (Table 3).

The effects of the F were significantly different on the AM spore numbers as shown by the 3-way ANOVA (Table 3). The short fallow recorded remarkably higher AM spore numbers than the forest fallow and cropping fields with respect to the 10–20 cm sampling depth of the Tko and Rku soils (Fig. 2g,i). The effects of D on the AM spore numbers were also significantly different (Table 3), with the 10–20 cm depth displaying higher AM spore numbers than the 0–10 cm depth with respect to the Rku soil (Fig. 2i). Additionally, greater AM spore numbers were observed in the 10–20 cm than in the 0–10 cm sampling depth of the Tko soil under the short fallow conditions (Fig. 2g). When comparing the AM spore numbers across the F, S and D, the values ranged from 49 to 1406 (Fig. 2g–i); with the 0–10 cm sampling depth of the Tku soil under the forest fallow displaying the lowest value, while the 10–20 cm depth of the Rku soil under the short fallow showing the highest value (Fig. 2h–i). None of the four tested interactions among the F, S and D was significant in terms of the AM spore numbers (Table 3).

### Relationships between the soil properties and AM community structure after the fallows and cropping

**Figure 3:**
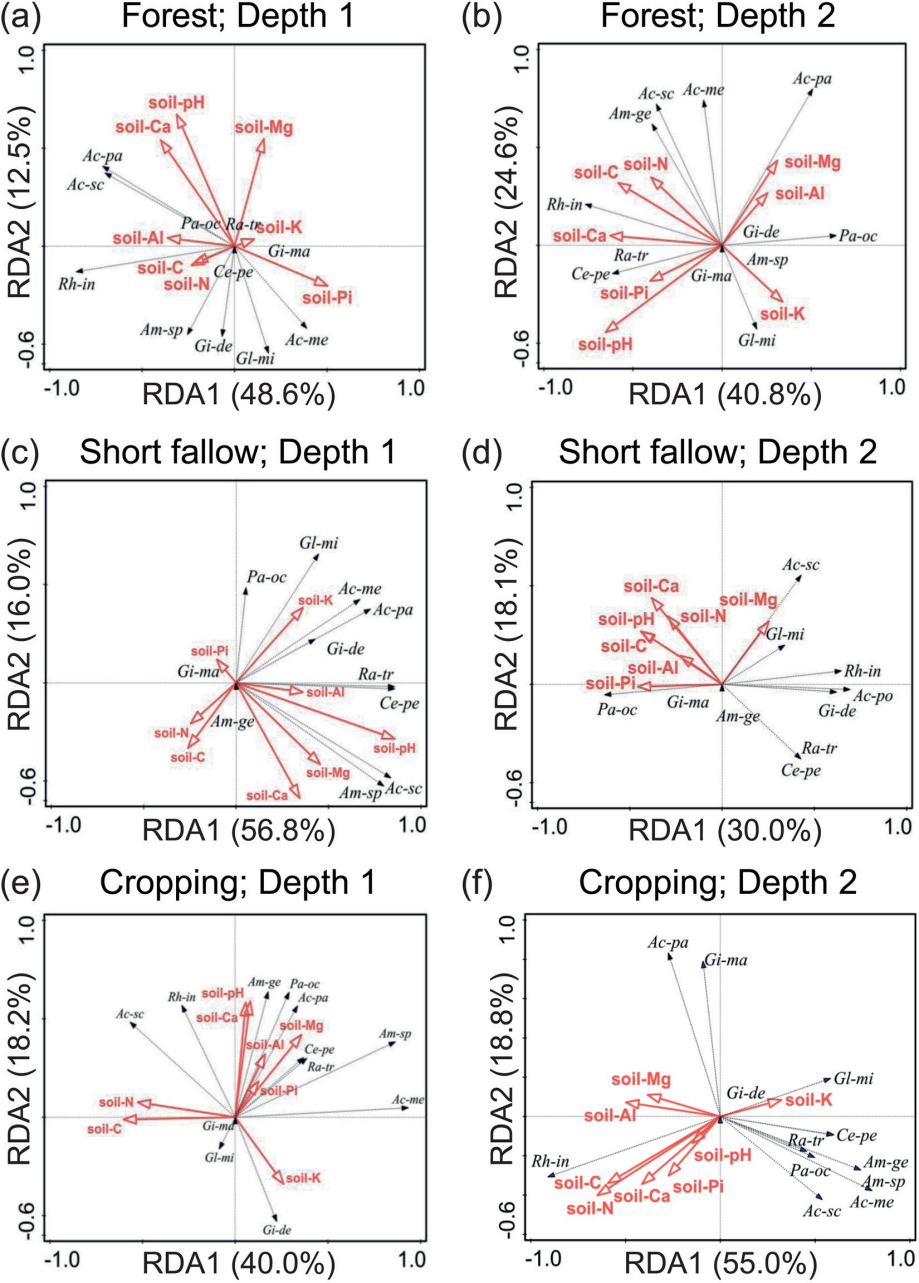
Biplots of the redundancy analysis (RDA) investigating the effects of the soil parameter predictors on the arbuscular mycorrhizal community composition by fallow types (forest fallow, short fallow and cropping conditions) and sampling depths (0–10 cm and 10–20 cm). (**a**,**b**) Forest fallow, (**c**,**d**) short fallow and (**e**,**f**) cropping fields with the two sampling depths (Depth 1, 0–10 cm; Depth 2, 10–20 cm). Soil-pH, pH measured in soil suspension; soil-N, nitrogen content; soil-C, carbon content; soil-Mg, magnesium content; soil-Al, aluminium content; soil-K, potassium content; soil-Ca, calcium content; soil-Pi, inorganic phosphate content in soil; *Ac-sc*, *Acaulospora scrobiculata*; *Ac-me*, *Acaulospora mellea*; *Ac-pa*, *Acaulospora paulineae*; *Pa-oc*, *Paraglomus occultum*; *Ce-pe*, *Cetraspora pellucida*; *Ra-tr*, *Racocetra tropicana*; *Gi-de*, *Gigaspora decipiens*; *Gi-ma*, *Gigaspora margarita*; *Gl-mi*, *Glomus microaggregatum*; *Rh-in*, *Rhizophagus intraradices*; *Am-ge*, *Ambispora gerdemannii*; *Am-sp*, *Ambispora* sp.

Irrespective of the fallow types (F) and sampling depths (D), eleven (*A*. *scrobiculata*, *A*. *mellea*, *A*. *paulineae*, *P*. *occultum*, *C*. *pellucida*, *R*. *tropicana*, *G*. *decipiens*, *G*. *margarita*, *G*. *microaggregatum*, *A*. *gerdemannii* and *Ambispora* sp.) of the 12 AM species displayed linear response curves along with the first DCA axis (DCA1), with non-linear curve being identified with *R*. *intraradices* (Supplementary Fig. 2). The DCA interpreting original variations extracted from the soil parameter predictors indicated that the longest DCA1 axes ranged from 39.8 to 49.3% of the AM species variabilities for the two sampling depths under the three forest fallow, short fallow and cropping conditions (Supplementary Fig. 3). Next, we performed a RDA to explain the relationships between the AM community structure and various soil characteristics (SC) under various combinations of D and F (Fig. 3). In the 0–10 cm soil depth of the forest fallow, the soil-pH and soil-Ca content exhibited significant influences on the AM community composition (Supplementary Table 2), whereas three (e.g. soil-Mg, -Al and -Ca contents) out of eight analyzed SC displayed significant roles in explaining the AM community composition in the 10–20 cm subsoil layer of the forest fallow (Supplementary Table 2). We further noticed remarkable contributions from soil-pH, -Mg and -K variables to the AM variability in the 0–10 cm depth under the short fallow (Supplementary Table 2), while none of the SC used as the predictors showed significant effect on the AM community composition in the 10–20 cm soil depth under the short fallow (Supplementary Table 2). The soil-Mg and -Ca contents displayed significant contributions to the variation in the AM community structure in the 0–10 cm soil depth of the cropping fields, while the soil-C and soil-Pi variables revealed their significant proportion in the AM composition variation in the 10–20 cm soil depth of the cropping fields (Supplementary Table 2). It should be noticed that among the *p*-adjusted values reporting the differential effects among the different soil parameters (soil-pH, -N, -C, -Mg, -Al, -K, -Ca or -Pi) on the AM species variabilities, only that of the soil-pH variable with respect to the 0–10 cm soil depth under the short fallow conditions was significant (Supplementary Table 2).

Table 4 depicts the correlation patterns between the individual soil property and the species abundance distribution (SAD) of each of the AM species under different soil depths, and fallow and cropping conditions. In the 0–10 cm soil depth of the forrest fallow, the relationships between the soil-pH and the SAD of *Ambispora* sp.; between the soil-Mg content and the SADs of *Ambispora* sp. and *C*. *pellucida*; and between the soil-Al content and the SAD of *A*. *scrobicultata* were significant. Furthermore, the soil-Ca content exhibited positive relationships with the SADs of *A*. *scrobiculata*, *A*. *paulineae* and *R*. *tropicana* in the 0–10 cm soil depth of the forest fallow. In the 10–20 cm soil layer of the forest fallow, both soil-pH and soil-Ca content showed significant correlations with the SADs of *C*. *pellucida* and *R*. *tropicana* (Table 4).

**Table 4:** Effects of various soil characteristics on the individual arbuscular mycorrhizal species in the two sampling depths (0–10 cm and 10–20 cm) under various fallow and cropping conditions.

	Depth 0–10 cm								Depth 10–20 cm							
	Soil-pH	Soil-N	Soil-C	Soil-Pi	Soil-Mg	Soil-Al	Soil-K	Soil-Ca	Soil-pH	Soil-N	Soil-C	Soil-Pi	Soil-Mg	Soil-Al	Soil-K	Soil-Ca
		[g kg^−1^]	[g kg^−1^]	[mg kg^−1^]	[cmol (+) kg^−1^]					[g kg^−1^]	[g kg^−1^]	[mg kg^−1^]	[cmol (+) kg^−1^]			
	**Forest fallow**															
*Acaulospora scrobiculata*	2.5 (0.13)	1.1 (0.37)	0.94 (0.57)	1.8 (0.22)	2.2 (0.16)	**4.3 (0.04)**	0.40 (0.64)	**9.0 (0.007)**	0.79 (0.51)	1.4 (0.30)	1.5 (0.27)	0.52 (0.60)	0.33 (0.73)	0.41 (0.67)	0.80 (0.52)	0.58 (0.57)
*Acaulospora mellea*	3.6 (0.08)	1.8 (0.21)	1.5 (0.27)	2.5 (0.13)	3.8 (0.06)	2.6 (0.12)	0.79 (0.52)	0.87 (0.55)	1.0 (0.38)	0.65 (0.54)	1.2 (0.34)	1.1 (0.38)	0.58 (0.57)	1.2 (0.33)	2.1 (0.17)	0.93 (0.57)
*Acaulospora paulineae*	3.1 (0.09)	0.65 (0.54)	1.0 (0.39)	2.0 (0.18)	3.1 (0.09)	4.2 (0.05)	0.35 (0.71)	**10.2 (0.004)**	2.1 (0.18)	0.27 (0.76)	0.68 (0.52)	3.4 (0.08)	1.3 (0.31)	2.2 (0.16)	2.1 (0.17)	1.1 (0.38)
*Ambispora* sp.	**6.6 (0.01)**	0.65 (0.54)	1.2 (0.34)	0.92 (0.56)	**6.6 (0.01)**	1.2 (0.34)	0.31 (0.73)	0.91 (0.56)	1.2 (0.35)	0.44 (0.35)	0.53 (0.60)	1.8 (0.22)	0.65 (0.54)	2.1 (0.17)	0.37 (0.69)	0.39 (0.68)
*Cetraspora pellucida*	1.8 (0.22)	0.44 (0.65)	0.45 (0.64)	0.72 (0.51)	**5.4 (0.02)**	0.53 (0.60)	0.30 (0.74)	0.87 (0.55)	**14.7 (0.001)**	1.9 (0.20)	1.9 (0.20)	0.75 (0.50)	0.34 (0.71)	0.78 (0.51)	1.1 (0.37)	**67.9 (<0.001)**
*Racocetra tropicana*	1.8 (0.21)	2.0 (0.18)	1.8 (0.22)	1.1 (0.36)	2.5 (0.14)	3.2 (0.08)	0.28 (0.75)	**8.1 (0.009)**	**12.2 (0.002)**	1.9 (0.30)	1.2 (0.34)	0.91 (0.56)	0.24 (0.78)	1.0 (0.39)	2.1 (0.17)	**22.4 (0.003)**
*Gigaspora decipiens*	0.83 (0.53)	0.51 (0.61)	0.70 (0.51)	1.2 (0.35)	0.83 (0.53)	1.0 (0.39)	0.28 (0.75)	0.09 (0.90)	0.96 (0.58)	3.3 (0.08)	2.7 (0.12)	2.4 (0.14)	2.0 (0.19)	3.1 (0.09)	0.90 (0.56)	1.7 (0.24)
*Glomus microaggregatum*	1.7 (0.24)	0.51 (0.61)	0.36 (0.70)	1.0 (0.39)	1.7 (0.24)	0.4 (0.66)	0.38 (0.69)	0.53 (0.60)	1.3 (0.32)	1.1 (0.38)	1.6 (0.24)	0.83 (0.53)	0.97 (0.58)	2.2 (0.17)	0.58 (0.57)	1.0 (0.39)
*Rhizophagus intraradices*	0.45 (0.64)	0.88 (0.55)	2.4 (0.13)	0.80 (0.52)	0.45 (0.64)	0.70 (0.52)	0.78 (0.51)	0.16 (0.84)	1.8 (0.22)	3.8 (0.06)	4.0 (0.05)	0.11 (0.89)	0.60 (0.56)	0.78 (0.51)	0.45 (0.64)	2.3 (0.15)
*Paraglomus occultum*	0.96 (0.58)	1.4 (0.29)	0.67 (0.53)	1.3 (0.32)	3.1 (0.09)	0.15 (0.85)	1.6 (0.26)	0.05 (0.94)	1.2 (0.35)	0.41 (0.67)	0.57 (0.58)	0.55 (0.59)	2.6 (0.12)	0.54 (0.59)	1.8 (0.22)	0.16 (0.85)
*Ambispora gerdemannii*	4.0 (0.05)	0.85 (0.54)	0.99 (0.57)	0.34 (0.71)	4.0 (0.05)	1.1 (0.38)	1.1 (0.37)	0.98 (0.59)	0.44 (0.65)	1.1 (0.36)	1.3 (0.31)	0.54 (0.44)	0.35 (0.70)	0.55 (0.59)	0.44 (0.65)	0.36 (0.70)
*Gigaspora margarita*	0.09 (0.90)	0.15 (0.86)	0.53 (0.60)	0.79 (0.51)	0.18 (0.83)	0.78 (0.51)	7.1 (0.01)	2.0 (0.19)	1.2 (0.35)	0.44 (0.65)	0.53 (0.60)	1.8 (0.22)	0.65 (0.54)	2.1 (0.17)	0.37 (0.69)	0.39 (0.68)
	**Short fallow**															
*Acaulospora scrobiculata*	**35.2 (<0.001)**	1.1 (0.37)	1. (0.35)	1.7 (0.24)	**7.1 (0.01)**	2.0 (0.19)	0.41 (0.67)	2.9 (0.10)	**8.6 (0.008)**	1.8 (0.22)	0.73 (0.50)	**5.3 (0.03)**	**10.2 (0.004)**	1.2 (0.35)	0.30 (0.74)	3.1 (0.09)
*Acaulospora mellea*	**4.8 (0.03)**	1.6 (0.25)	2.8 (0.11)	2.0 (0.19)	**4.8 (0.03)**	1.5 (0.27)	1.5 (0.28)	0.82 (0.53)	3.2 (0.09)	2.0 (0.18)	3.6 (0.06)	2.3 (0.15)	1.1 (0.37)	0.66 (0.52)	3.9 (0.05)	**4.7 (0.03)**
*Acaulospora paulineae*	2.0 (0.19)	1.4 (0.29)	1.5 (0.27)	0.40 (0.68)	0.62 (0.55)	0.61 (0.56)	3.2 (0.08)	2.5 (0.13)	0.79 (0.51)	**8.3 (0.009)**	**6.3 (0.01)**	0.83 (0.53)	0.57 (0.58)	0.69 (0.52)	0.61 (0.56)	0.84 (0.53)
*Ambispora* sp.	**27.4 (<0.001)**	0.92 (0.56)	0.99 (0.59)	1.9 (0.20)	**6.1 (0.02)**	1.9 (0.21)	0.33 (0.72)	2.7 (0.11)	0.34 (0.71)	1.5 (0.26)	0.71 (0.51)	0.87 (0.55)	0.38 (0.69)	0.80 (0.52)	0.61 (0.56)	0.68 (0.52)
*Cetraspora pellucida*	**4.5 (0.04)**	0.81 (0.52)	0.87 (0.54)	1.5 (0.26)	1.1 (0.37)	1.1 (0.36)	0.29 (0.75)	1.8 (0.21)	0.34 (0.71)	0.48 (0.62)	0.49 (0.62)	0.44 (0.55)	1.1 (0.37)	0.32 (0.73)	2.1 (0.18)	0.58 (0.57)
*Racocetra tropicana*	**4.6 (0.04)**	0.84 (0.53)	0.90 (0.56)	1.4 (0.27)	1.3 (0.33)	1.1 (0.36)	0.31 (0.70)	1.4 (0.19)	0.30 (0.74)	0.82 (0.52)	0.36 (0.70)	0.25 (0.78)	0.46 (0.64)	0.58 (0.57)	0.74 (0.50)	0.34 (0.71)
*Gigaspora decipiens*	2.5 (0.13)	0.25 (0.77)	0.38 (0.69)	0.75 (0.50)	0.45 (0.64)	0.69 (0.52)	29.6 (<0.001)	1.0 (0.39)	0.45 (0.64)	0.75 (0.50)	0.62 (0.55)	0.39 (0.68)	2.3 (0.15)	0.50 (0.62)	0.68 (0.52)	0.58 (0.57)
*Glomus microaggregatum*	**7.9 (0.01)**	0.72 (0.51)	1.1 (0.36)	2.0 (0.18)	0.46 (0.63)	0.22 (0.80)	3.7 (0.06)	1.5 (0.27)	**5.2 (0.03)**	0.73 (0.50)	0.45 (0.64)	1.5 (0.27)	**5.0 (0.03)**	0.38 (0.69)	0.78 (0.51)	1.2 (0.34)
*Rhizophagus intraradices*	0.89 (0.55)	2.6 (0.13)	1.8 (0.21)	1.3 (0.31)	0.58 (0.57)	0.72 (0.50)	2.1 (0.17)	**14.2 (<0.001)**	0.44 (0.65)	**9.5 (0.006)**	**8.8 (0.007)**	0.36 (0.70)	0.37 (0.69)	1.6 (0.25)	0.26 (0.77)	0.24 (0.78)
*Paraglomus occultum*	0.80 (0.52)	0.54 (0.59)	0.67 (0.53)	0.67 (0.53)	1.2 (0.33)	0.92 (0.57)	0.34 (0.71)	1.7 (0.23)	3.8 (0.06)	1.4 (0.29)	1.9 (0.20)	3.5 (0.07)	0.72 (0.51)	0.92 (0.56)	0.72 (0.51)	2.8 (0.11)
*Ambispora gerdemannii*	1.4 (0.29)	0.66 (0.53)	0.96 (0.58)	0.48 (0.62)	1.0 (0.39)	0.30 (0.74)	4.1 (0.05)	1.8 (0.21)	0.77 (0.51)	1.0 (0.39)	0.79 (0.51)	0.71 (0.51)	0.79 (0.51)	1.2 (0.34)	2.0 (0.19)	0.41 (0.67)
*Gigaspora margarita*	1.1 (0.38)	0.93 (0.57)	1.3 (0.32)	0.79 (0.51)	0.94 (0.57)	0.36 (0.70)	3.0 (0.10)	1.2 (0.34)	3.0 (0.10)	0.58 (0.57)	1.5 (0.26)	1.7 (0.23)	0.32 (0.53)	**5.1 (0.03)**	0.32 (0.72)	1.5 (0.27)
	**Cropping**															
*Acaulospora scrobiculata*	0.56 (0.58)	0.55 (0.59)	0.67 (0.53)	1.7 (0.24)	**8.3 (0.008)**	2.9 (0.10)	0.84 (0.53)	0.31 (0.73)	2.0 (0.19)	0.90 (0.56)	0.40 (0.67)	0.10 (0.89)	0.62 (0.55)	1.7 (0.23)	0.51 (0.61)	1.6 (0.25)
*Acaulospora mellea*	0.57 (0.58)	2.4 (0.14)	**4.7 (0.03)**	1.2 (0.33)	1.2 (0.34)	0.41 (0.67)	0.78 (0.51)	0.73 (0.50)	0.72 (0.52)	2.7 (0.12)	2.0 (0.16)	0.57 (0.58)	039 (0.68)	2.1 (0.18)	1.1 (0.36)	1.2 (0.33)
*Acaulospora paulineae*	3.0 (0.08)	0.52 (0.61)	1.2 (0.34)	3.0 (0.10)	**7.4 (0.01)**	2.2 (0.17)	3.1 (0.09)	**11.9 (0.002)**	0.33 (0.72)	**6.4 (0.01)**	2.1 (0.17)	0.74 (0.50)	0.61 (0.56)	0.37 (0.69)	0.38 (0.69)	0.99 (0.59)
*Ambispora* sp.	0.15 (0.85)	0.64 (0.54)	3.3 (0.08)	0.31 (0.74)	1.0 (0.39)	1.7 (0.23)	0.35 (0.71)	0.47 (0.63)	0.92 (0.57	**5.0 (0.03)**	**5.5 (0.02)**	1.8 (0.21)	0.58 (0.57)	3.3 (0.08)	1.4 (0.28)	2.4 (0.14)
*Cetraspora pellucida*	0.68 (0.52)	1.6 (0.26)	**7.2 (0.01)**	0.57 (0.58)	0.24 (0.74)	1.1 (0.35)	0.15 (0.86)	0.19 (0.82)	0.98 (0.58)	2.8 (0.11)	2.9 (0.10)	1.9 (0.19)	1.7 (0.24)	0.85 (0.54)	4.0 (0.05)	1.1 (0.36)
*Racocetra tropicana*	0.44 (0.63)	1.2 (0.34)	**5.9 (0.04)**	0.52 (0.60)	0.40 (0.68)	1.2 (0.35)	0.15 (0.86)	0.15 (0.85)	0.56 (0.58)	2.4 (0.14)	2.0 (0.18)	1.2 (0.34)	1.3 (0.32)	0.65 (0.54)	2.8 (0.11)	0.64 (0.54)
*Gigaspora decipiens*	1.5 (0.27)	0.55 (0.59)	0.37 (0.69)	**5.5 (0.02)**	1.5 (0.27)	0.60 (0.58)	0.41(0.67)	0.91 (0.56)	2.4 (0.14)	0.52 (0.60)	0.51 (0.61)	0.19 (0.82)	1.5 (0.26)	0.32 (0.74)	0.47 (0.63)	0.62 (0.55)
*Glomus microaggregatum*	0.96 (0.58)	2.7 (0.12)	**4.6 (0.04)**	0.44 (0.65)	1.3 (0.33)	0.62 (0.55)	2.3 (0.15)	1.5 (0.26)	2.0 (0.19)	4.0 (0.05)	**4.3 (0.04)**	4.2 (0.05)	0.59 (0.57)	2.0 (0.18)	1.9 (0.19)	1.6 (0.24)
*Rhizophagus intraradices*	0.73 (0.50)	0.79 (0.52)	1.6 (0.25)	0.3 (0.73)	2.1 (0.17)	**7.2 (0.01)**	2.9 (0.10)	1.1 (0.38)	0.51 (0.61)	1.5 (0.27)	3.4 (0.08)	**5.9 (0.02)**	0.49 (0.62)	0.91 (0.54)	**5.2 (0.03)**	3.0 (0.09)
*Paraglomus occultum*	3.0 (0.08)	0.87 (0.54)	1.6 (0.25)	2.4 (0.14)	4.2 (0.05)	2.0 (0.19)	2.7 (0.12)	**5.4 (0.02)**	0.28 (0.75)	0.67 (0.53)	0.60 (0.55)	0.25 (0.77)	0.74 (0.50)	**21.5 (0.003)**	0.80 (0.52)	2.9 (0.10)
*Ambispora gerdemannii*	1.3 (0.33)	0.98 (0.59)	1.4 (0.29)	3.3 (0.08)	3.4 (0.08)	0.97 (0.58)	**6.5 (0.01)**	**7.4 (0.01)**	3.0 (0.10)	0.67 (0.53)	0.70 (0.52)	0.27 (0.76)	0.66 (0.53)	0.20 (0.81)	0.87 (0.55)	0.36 (0.70)
*Gigaspora margarita*	2.1 (0.17)	0.30 (0.71)	0.93 (0.57)	1.5 (0.28)	3.1 (0.09)	3.2 (0.09)	**6.1 (0.02)**	3.6 (0.06)	0.26 (0.77)	**7.5 (0.01)**	2.8 (0.11)	1.3 (0.31)	0.63 (0.55)	0.31 (0.73)	0.89 (0.49)	1.7 (0.22)

The *f*-values displaying correlation levels between the pairs of variables are shown, with the respective *p*-value in parentheses. Values in bold letters indicate significant *f*-values between the pairs of variables at *p* < 0.05.

As for the short fallow conditions, the SADs of six out 12 AM species, namely *A*. *scrobicultata*, *A*. *mellea*, *Ambispora* sp., *C*. *pellucida*, *R*. *tropicana* and *G*. *microagregatum* were significantly related to the soil-pH in the 0–10 cm soil depth (Table 4). Similarly, the SADs of *A*. *scrobiculata*, *A*. *mellea* and *Ambispora* sp. and the soil-Mg content were significantly interrelated; while that of *G*. *decipiens* and the soil-K content, and that of *R*. *intraradices* and the soil-Ca content showed a positive correlation in the 0–10 cm soil depth of the short fallow. In comparing the relationships between the soil properties and the SAD of the AM fungi in the 10–20 cm soil depth under the short fallow conditions, we found that both soil-pH and soil-Mg content exhibited significant correlations with the SADs of *A*. *scrobiculata* and *G*. *microagregatum*, while both soil-N and -C contents showed significant relationships to the SADs of *A*. *paulineae* and *R*. *intraradices*. Furthermore, significant relationships were also observed between soil-Pi content and the SAD of *A*. *scrobiculata*; soil-Al content and the SAD of *G*. *margarita* and; and soil-Ca content and the SAD of *A*. *mellea* in the 10–20 cm soil depth under the short fallow conditions (Table 4).

With regard to the cropping fields, the soil-C content displayed significant relationships with the SADs of *A*. *mellea*, *C*. *pellucida*, *R*. *tropicana* and *G*. *microagregatum* in the 0–10 cm depth, while the soil-Mg content exhibited significant correlations with the SADs of *A*. *scrobiculata* and *A*. *paulineae* (Table 4). The soil-Ca content was significantly related to the SADs of *A*. *paulineae*, *P*. *ocultum* and *A*. *gerdermannii*, whereas the soil-K content showed significant relationships to the SADs of *A*. *gerdermannii* and *G*. *margarita* in the 0–10 cm sampling depth of the cropping fields. Significant correlations were also observed between soil-Pi content and *G*. *decipiens*, and between soil-Al content and *R*. *intraradices* under the same conditions. After the cropping, in the 10–20 cm soil depth, the soil-Pi content showed significant correlation with the SAD of *R*. *intraradices*, while the soil-N content exhibited significant relationships with the SADs of *A*. *paulineae*, *Ambispora* sp. and *G*. *margarita*. Furthermore, we recorded significant relationships between the soil-C content and the SADs of *Ambispora* sp. and *G*. *microaggregatum*, soil-Al content and the SAD of *P*. *occultum*, and soil-K content and the SAD of *R*. *intraradices* in the 10–20 cm soil depth of the cropping fields (Table 4).

### Identification of factors altering the AM community composition after the fallows and cropping

**Figure 4:**
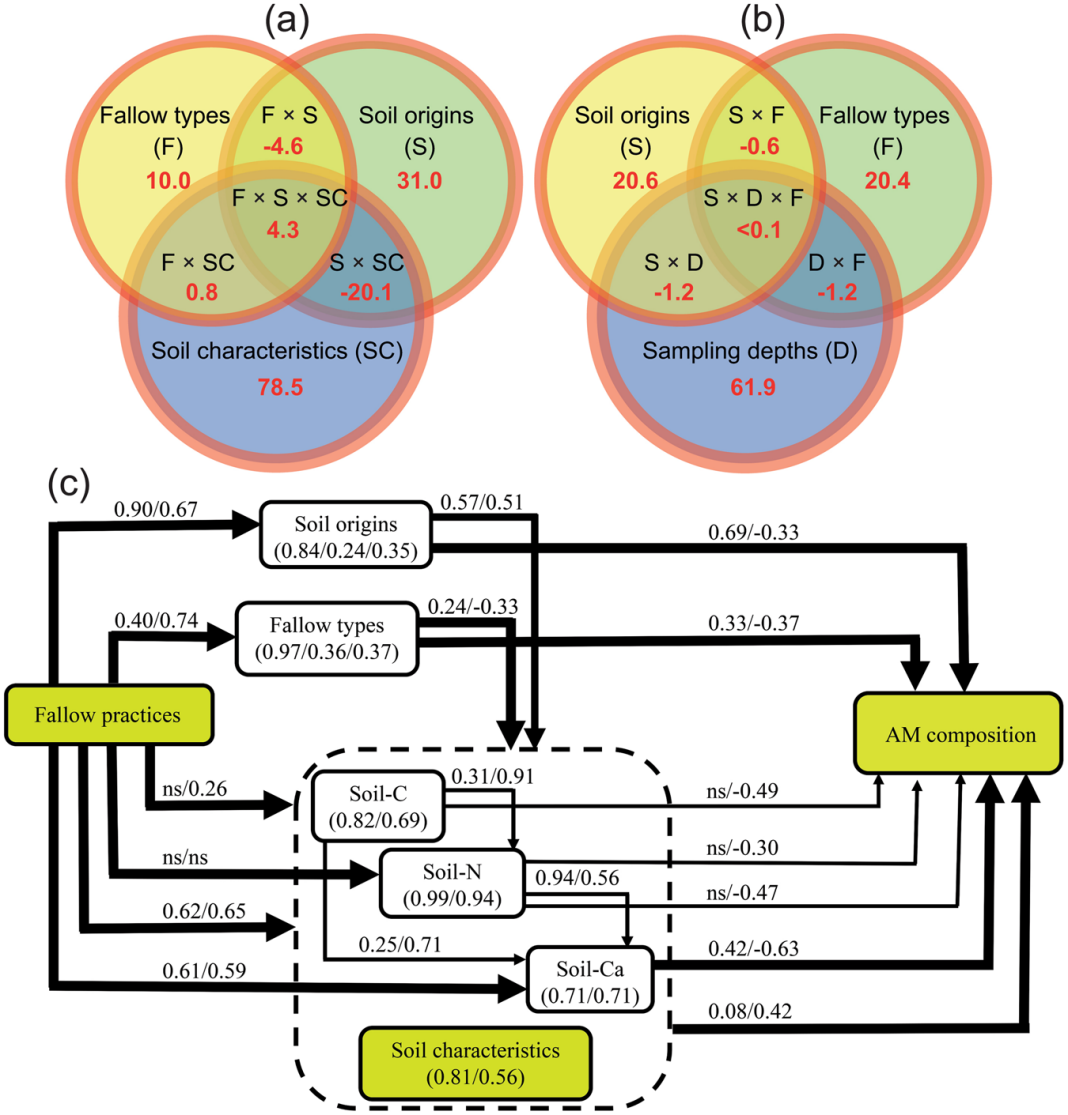
Venn diagrams of the RDA-based variation-partitioning results indicating the proportions (in percentage, %) from (**a**) the quantitative and factor estimators, fallow types (F, three levels; forest fallow, short fallow and cropping conditions), soil origins (S, three levels; *Typic Kandiudox*, *Typic Kandiudult* and *Rhodic Kandiudult*) and soil charactrisitics (SC, eight parmeters; soil-pH, pH measured in soil suspension; soil-N, nitrogen content; soil-C, carbon content; soil-Mg, magnesium content; soil-Al, aluminium content; soil-K, potassium content; soil-Ca, calcium content; and soil-Pi, inorganic phosphate content measured in soil), and their combinations; and from (**b**) the quantitative and factor estimators S, F and sampling depths (D, two levels; 0–10 cm and 10–20 cm), and their combinations on the arbuscular mycorrhizal (AM) community composition. (**c**) Multigroup structural equation modeling displaying the pathways by which fallow practices showed influences on the soil properties and the AM community composition in two soil depths (0–10 cm/10–20 cm). The various pathways drawn from the models used the dataset as single group. Path coefficient values shown above to the pathways are for the soil depths and listed in their order (0–10 cm/10–20 cm). Only the significant path coefficient values (*p* < 0.05) from the models are presented. The thick and thin arrows indicate the direct and indirect effects, respectively. Dotted frame indicates the soil properties, namely soil-C, -N and -Ca contents that strongly influenced the model groups. Values in parentheses indicate path coefficient values for the main models using the dataset as single group. *ns*, not significant.

Results of the RDA-based variation-partitioning approach dissecting the contribution of each of the predictors on the AM community composition revealed that the soil characteristics (SC), soil origins (S) and fallow types (F) were responsible for 78.5, 31.0 and 10.0% of the variations in the AM community composition, respectively (Fig. 4a). Among the SC, soil-pH, and soil-Ca, -Mg, -C and -N contents exhibited significant effects on the variations of the AM composition structure across the sampled locations (Supplementary Table 3). The *p*-adjusted values explained the significant variations for the soil-pH, and soil-Ca, -Mg, -C and -N contents among the SC (Supplementary Table 3). Among the various combinations of F, S and SC, the combination S × SC had the lowest effect on the variation of the AM composition structure as factorial predictor, followed by the F × S (Fig. 4a). In analyzing the influences of the factorial F, S and D predictors and their combinations, we found that the D, S and F were responsible for 61.9, 20.6 and 20.4% of the variations in the AM community structure, respectively, while their combinations F × S, F × D, S × D, F × S × D exhibited neglible effects on the AM community composition structure variability (Fig. 4b). These data indicated that the effects of the individual predictors were stronger than that of their combinations in controlling the AM species composition and distribution under the analyzed environmental conditions (Fig. 4a,b).

Results of the SEM using the data of analyzed soil properties under the different F, S and D conditions as a single group supported our previous observations obtained from the RDA, and further indicated the different pathways and effects in which each predictor exercised its influence on the AM community composition (Fig. 4c). The final built models fitted well to our original data with a non-significant χ^2^ of 52.3, a SRMR value < 0.08 and a CFI value > 0.9, indicating that the hypothesized measurement models properly fitted to the data. Soil properties included as latent variables in the models indicated their direct effects on the AM composition, with the measured soil-C, -N and -Ca variables significantly connecting the soil properties and AM composition (Fig. 4c). The fallow practices, when included in the model, also showed direct effects on soil characteristics, with the measured soil-C, -N and -Ca variables displaying strong relationships to the fallow practices on the basis of the observed path coefficients (Fig. 4c). On the other hand, we discovered that the fallow practices exhibited indirect influences on the AM composition (Fig. 4c). When the models were constructed separately using the datasets obtained from the two soil depths, we found that the fallow practices and soil characteristics disclosed direct effects on each other, and the soil-Ca variable exhibited relatively strong relationships to fallow practices under both sampling depths (Fig. 4c). The soil origins showed strong direct effects on the AM community compositions in the upper 0–10 cm soil depth only (Fig. 4c). With respect to the fallow types, they exhibited indirect weak effects on the AM composition under both sampling depths as observed from their path coefficients (Fig. 4c).

## Discussion

### Changes in soil characteristics by fallow types across soil origins and sampling depths

In this study, we first dissected the contributions of the fallow types (F), including forest fallow, short-fallow and cropping to the changes in chemical characteristics of three soil types (S; Tko, Tku and Rku) in the two sampling depths (D; 0–10 and 10–20 cm). Our results demonstrated a clear tendency that the examined F exhibited significantly different effects on the chemical properties of all three types of soils collected from the two sampling depths (Tables 1 and 2). Additionally, the S and D significantly impacted the analyzed soil parameters (Tables 1 and 2). Remarkable differences in soil-pH of different soil types have been reported earlier in the soils sampled in these areas of southern Cameroon^39^. Similarly, significant differences among the soil types regarding the available soil-Pi content were also previously noted, when the earthworm cast production was examined in different forest and short fallow fields at the same locations^39^. The deterioration in available soil-Pi content in the cropping fields as compared with the forest fallow fields was observed in the Tko soils for both sampling depths (Table 2), corroborating the hypothesis of nutrient deterioration due to cropping which is partly restored through long-term fallowing process under shifting cultivation system^5^. The PCA approach allowed us to summarize the different soil data into two orthogonal components from the various F, S and D conditions (Fig. 1; Supplementary Table 1). The first PC component showed highly positive loading scores for soil-pH and -Ca in the 0–10 cm soil depth of the forest fallow and cropping conditions, and in the 10–20 cm soil depth under the short fallow and cropping conditions, irrespective of the S (Supplementary Table 1). It is worth mentioning that the Rku soil showed a closer relationship with soil-pH than Tko and Tku soils in both two soil depths under all three forest fallow, short fallow and cropping conditions (Fig. 1), which might be associated with the higher soil-Ca content observed in the Rku than in Tko and Tku soils (Fig. 1; Table 2).

### Effects of fallow types, soil origins and sampling depths on the AM species richness, diversity and abundance

In the present study, we recorded a maximum of 12 AM species from the two sampling depths (D) of three different soil types (S) under two fallow and a cropping conditions. The observed AM diversity is in accordance with results of other investigations conducted in Kenya^55^, China^56^, Benin^57^ and Switzerland^58^. On the other hand, a number of studies have noticed up to 30 AM species cohabiting in the fields of the temperate climate regions^25,59,60^. Possible reasons of the relatively low AM diversity observed in the present study could be attributed to the use of the trap culture approach that could potentially inhibit the sporulation of rare AM species, and thus might underestimate the α-diversity^61^. We opted to use the trap culture method to recover fresh AM spores that were further used as isolates for AM inoculant production for the planned assays in the frame of this project. Another possible reason of the low AM diversity could be due to the incompatibilities between the primers used and the target regions, which might result in low matching of the original sequence and lack of possibility to detect some important rare AM species^62^. In addition, the limited availability of reference sequence data for various AM species could contribute to the reduction in recovery of more AM species.

We further noticed that the species abundance distributions (SADs) of several AM species, mainly *A*. *scrobiculata*, *A*. *mellea*, *Ambispora* sp. and *R*. *intraradices*, were greatly influenced by the F (Table 3), with the cropping revealing the highest AM species richness (Fig. 2a–f). The effects of the cropping practices, such as tillage, crop rotation and other agricultural management approaches, on the AM community compositions have been extensively studied^5,25,32,63^. Cropping practices comprising of tillage, crop rotational systems, and/with organic manuring incorporation and inorganic fertilizer applications negatively affect the AM community composition, and lead to shifts in composition and reduction in the AM diversity^16,25,64^. In addition, the effects of the S were found to be significant on the SADs of several AM species, particularly *A*. *mellea*, *Ambispora* sp., *C*. *pellucida*, *R*. *tropicana* and *G*. *microaggregatum* (Table 3). This result is supported by a previous report that showed that S is among the determinant factors for the AM diversity in the Swiss agroecosystem^58^.

To explore the extent to which top and subsoil layers influence the AM species richness and diversity in a community under different fallow and cropping conditions, we conducted different hierarchical analyses. We found that the D remarkably impacted the AM species richness and Shannon-Weaver diversity index (Table 3). Interestingly, the subsoil layer (10–20 cm) displayed higher AM species richness and Shannon-Weaver diversity index than the topsoil layer (0–10 cm) under all fallow and cropping conditions of the Rku soils (Fig. 2c,f). Recent studies conducted under the temperate climate conditions have also shown different impacts of the topsoil and subsoil layers on the species richness and Shannon-Weaver diversity index of the AM fungi^32,65^. Factors driving the AM community compositions across the different fallow and cropping conditions, as well as the sampling soil layers remain to be determined, particularly in the tropical soils. Plausible hypotheses are linked to the differences in the composition of native plant species used, which play an important role in shaping the AM community composition in different soil depths as suggested by previous reports^32,65^. The reason that the cropping practice revealed higher AM species richness than both the forest and short fallows in almost all combinations of D and S still remains unknown as well (Fig. 2a–c).

### Factors influencing the AM community composition

Using the RDA-based variation-partitioning method and the SEM approach, we identified predictors of importance in structuring the AM community composition (Figs 3 and 4). Under different fallow type (F) and sampling depth (D) conditions, several members of the examined soil characteristics (SC) were shown to affect the AM community composition. The effect of soil-pH on the AM composition was particularly observed in the topsoil layer (0–10 cm) under the forest and short fallow conditions (Fig. 3a,c; Supplementary Table 2). Previous studies investigating the effects of soil-pH on the AM and bacterial community compositions suggested that low soil-pH conditions alter the microbe community structure^66,67^. Furthermore, the variation-partitioning method allowed us to define the contributions from different SC, F, soil origins (S) and D, as well as their combinations, to the AM composition variations (Fig. 4a,b). Among the SC, F and S, the SC and the F had the highest (78.5%) and lowest (10.0%) contribution, respectively, to the AM variation (Fig. 4a). A significant contribution of SC to the variation of AM community composition has also been reported in a previous investigation of soils on the North American Great Plains^32^. Furthermore, among the soil properties examined, the soil-pH, and soil-Ca, -Mg, -C, and -N contents showed significant effects on the AM composition structure (Supplementary Table 3). For instance, we observed that the soil-C content was highly related to the SADs of four out of 12 AM species [e.g. *A*. *mellea*, *C*. *pellucida*, *R*. *tropicana* and *G*. *microaggregatum* (Table 4)] in the topsoil layer under cropping conditions, suggesting a promoting role of the organic-C for growth and development of both plants and soil microorganisms^68,69^.

Previous investigations have demonstrated that the labile C fractions in soils provide energy source for soil microorganisms, and the proportion of organic matter in these fractions is a valuable indicator as for how the soils are biologically fertile^70^. Our findings are in agreement with the results of previous reports that showed that the community compositions of soil microbes, including bacteria and AM fungi, are under strong influences of the soil organic constituents^23,71,72^. Recent evidence has depicted the role of mycelia from AM fungi in decomposing the organic matter via the release of compounds like ammonia oxidizers, and secondary metabolites like phenolic compounds from the fungi to produce organic N for their uptake and utilization^23,72^. In addition, among the factorial predictors F, S and D, the D accounted for the highest variation of the AM community composition (61.9%) (Fig. 4b). These results are in agreement with recent observations that also indicated the influences of D on both the AMF species richness and community composition^32,65^.

Results from the SEM displayed various pathways in which each predictor exercised its effect (direct/indirect) on the AM community composition (Fig. 4c). The direct effect of the soil characteristics (SC) on structuring the AM community composition is consistent with previous observation^58^. The effects of fallow practices on the AM community compositions were indirect, while they were direct on the SC, with the observed strong path coefficients from the soil-C, -N and –Ca variables (Fig. 4c), suggesting that the fallow practices might affect the AM diversity by modifying the soil chemical properties, particularly the soil-C, -N and –Ca contents, to impose their influence on the surrounding microbial communities^32^. Interestingly, although soil-pH did not show a strong direct path from the model built by the SEM, it showed significant relationship with the AM community compositions in 0–10 cm soil depth under the forest and short fallows according to the results of RDA (Fig. 3; Supplementary Table 3).

In conclusion, the present study showed the differential effects of the F, S and D, and their interactions on the soil properties. Furthermore, the F, S and D, and their interactions also significantly and differently affected the AM species richness and diversity, with the cropping practices revealing the highest effect, supporting the hypothesis of a positive feedback of the cropping practices on the AM structures under the conditions of the shifting agriculture in tropical Africa. Our findings also suggest that the fallow practices indirectly affect the AM diversity by altering the soil chemical properties via the production of high amount of C and N, which will subsequently influence the development of different AM species in soils.

## Electronic supplementary material

Supplementary information
