# Supplementary material for: Mycorrhizal fungal community structure in tropical humid soils under fallow and cropping conditions
Source: Sci Rep. 2018 Nov 20;8:17061. doi: 10.1038/s41598-018-34736-6 (PMC6244078; doi:10.1038/s41598-018-34736-6)
Supplement: Supplementary file 1 — Supplementary information [file 41598_2018_34736_MOESM1_ESM.pdf]

**Supplementary information**

# Title: Mycorrhizal fungal community structure in tropical humid soils under fallow and cropping conditions

Authors: Martin Jemo, Driss Dhiba, Abeer Hashem, Elsayed Fathi Abd_Allah, Abdulaziz A. Alqarawi, Lam-Son Phan Tran

**Supplementary Table 1**. Principal component (PC) loading scores for different examined soil parameters in two sampling depths (0−10 cm and 10−20 cm) under the forest fallow, short fallow and cropping conditions. Data from three soil origins (*Typic Kandiudox*, *Typic Kandiudult* and *Rhodic Kandiudult*) were combined and used in the analysis. Soil-pH, pH measured in water solution; soil-N, nitrogen content; soil-C, carbon content; soil-Mg, magnesium content; soil-Al, aluminium content; soil-K, potassium content; soil-Ca, calcium content; soil-Pi, inorganic phosphate content in soil.

| | PC1 | PC2 | PC1 | PC2 |
|---|---|---|---|---|
| | Soil depth 0−10 cm | | Soil depth 10−20 cm | |
| | Forest fallow | | | |
| Soil-pH | 0.83 | 0.38 | 0.67 | -0.66 |
| Soil-N content (g $kg^{-1}$) | -0.52 | 0.78 | 0.82 | 0.46 |
| Soil-C content (g $kg^{-1}$) | -0.51 | 0.73 | 0.92 | 0.20 |
| Soil-Mg content [cmol (+) $kg^{-1}$] | -0.59 | -0.02 | 0.21 | 0.93 |
| Soil-Al content [cmol (+) $kg^{-1}$] | 0.07 | 0.72 | -0.10 | 0.93 |
| Soil-K content [cmol (+) $kg^{-1}$] | 0.48 | 0.58 | -0.57 | 0.13 |
| Soil-Ca content [cmol (+) $kg^{-1}$] | 0.85 | 0.16 | 0.87 | 0.09 |
| Soil-Pi content (mg $kg^{-1}$) | -0.73 | 0.003 | 0.84 | -0.26 |
| Eigen value | 3.10 | 2.1 | 3.8 | 2.5 |
| Total variance explained (TVE, %) | 38.7 | 27.6 | 48.2 | 31.9 |
| | Short fallow | | | |
| Soil-pH | 0.47 | 0.67 | 0.94 | -0.27 |
| Soil-N content (g $kg^{-1}$) | 0.58 | -0.77 | 0.78 | 0.53 |
| Soil-C content (g $kg^{-1}$) | 0.46 | -0.81 | 0.91 | 0.19 |
| Soil-Mg content [cmol (+) $kg^{-1}$] | 0.83 | 0.42 | 0.13 | 0.85 |
| Soil-Al content [cmol (+) $kg^{-1}$] | 0.82 | -0.04 | 0.81 | 0.81 |
| Soil-K content [cmol (+) $kg^{-1}$] | 0.60 | -0.01 | -0.34 | -0.34 |
| Soil-Ca content [cmol (+) $kg^{-1}$] | 0.62 | 0.62 | 0.97 | -0.10 |
| Soil-Pi content (mg $kg^{-1}$) | -0.55 | 0.33 | 0.79 | -0.51 |
| Eigen value | 3.2 | 2.4 | 4.6 | 2.2 |
| Total variance explained (TVE, %) | 40.0 | 29.8 | 58.0 | 27.3 |
| | Cropping | | | |
| Soil-pH | 0.85 | 0.27 | 0.83 | -0.32 |
| Soil-N content (g $kg^{-1}$) | 0.36 | -0.86 | 0.73 | 0.43 |
| Soil-C content (g $kg^{-1}$) | 0.30 | -0.90 | 0.91 | 0.23 |
| Soil-Mg content [cmol (+) $kg^{-1}$] | 0.94 | 0.20 | 0.02 | 0.85 |
| Soil-Al content [cmol (+) $kg^{-1}$] | 0.68 | 0.50 | 0.66 | -0.21 |
| Soil-K content [cmol (+) $kg^{-1}$] | -0.28 | 0.78 | 0.25 | -0.47 |
| Soil-Ca content [cmol (+) $kg^{-1}$] | 0.98 | 0.06 | 0.86 | 0.20 |

| | | | | |
|---|---|---|---|---|
| Soil-Pi content (mg $kg^{-1}$) | 0.69 | -0.04 | 0.93 | -0.21 |
| Eigen value | 3.8 | 2.5 | 4.2 | 1.4 |
| Total variance explained (TVE, %) | 47.9 | 32.0 | 52.8 | 18.0 |

**Supplementary Table 2.** Effects of different individual soil chemical parameters as predictors on arbuscular mycorrhizal community distribution under various combinations of sampling depths (0–10 cm and 10–20 cm), and forest fallow, short fallow and cropping conditions. Data from three soil origins (*Typic Kandiudox*, *Typic Kandiudult* and *Rhodic Kandiudult*) were combined and used in the analysis. Reported significances for the *f*-values were determined based on the Monte Carlo permutation with 499 permutations test. Asterisks indicate statistical significances. $*p < 0.05$; *ns*, not significant ($p \geq 0.05$). *p* (adjusted) values report the significance for the multiple comparisons among the different soil chemical parameters analyzed on the basis of false discovery rate.

| | Explained (%) | *f*-value | *p*-value | *p*-adjusted | Explained (%) | *f*-value | *p*-value | *p*-adjusted |
|---|---|---|---|---|---|---|---|---|
| | Soil depth 0–10 cm | | | | Soil depth 10–20 cm | | | |
| | Forest fallow | | | | | | | |
| Soil-pH | 15.8 | 1.8 | 0.04 (*) | 0.26 (*ns*) | 15.3 | 1.8 | 0.10 (*ns*) | 0.16 (*ns*) |
| Soil-N content (g $kg^{-1}$) | 11.6 | 1.5 | 0.24 (*ns*) | 0.48 (*ns*) | 6.9 | 0.8 | 0.13 (*ns*) | 0.6 (*ns*) |
| Soil-C content (g kg-$^{1}$) | 4.8 | 0.6 | 0.69 (*ns*) | 0.78 (*ns*) | 14.2 | 1.8 | 0.10 (*ns*) | 0.17 (*ns*) |
| Soil-Mg content [cmol (+) $kg^{-1}$] | 11.5 | 1.6 | 0.18 (*ns*) | 0.48 (*ns*) | 21.5 | 3.5 | 0.01 (*) | 0.07 (*ns*) |
| Soil-Al content [cmol (+) $kg^{-1}$] | 4.8 | 0.6 | 0.67 (*ns*) | 0.78 (*ns*) | 11.4 | 3.0 | 0.02 (*) | 0.07 (*ns*) |
| Soil-K content [cmol (+) $kg^{-1}$] | 5.9 | 0.8 | 0.52 (*ns*) | 0.78 (*ns*) | 2.8 | 0.8 | 0.53 (*ns*) | 0.60 (*ns*) |
| Soil-Ca content [cmol (+) $kg^{-1}$] | 15.0 | 2.0 | 0.04 (*) | 0.26 (*ns*) | 14.9 | 3.0 | 0.02 (*) | 0.07 (*ns*) |
| Soil-Pi content (mg $kg^{-1}$) | 2.7 | 0.3 | 0.87 (*ns*) | 0.87 (*ns*) | 6.7 | 2.1 | 0.11 (*ns*) | 0.17 (*ns*) |
| | Short fallow | | | | | | | |
| Soil-pH | 25.6 | 3.4 | 0.02 (*) | 0.01 (*) | 7.5 | 0.9 | 0.46 (*ns*) | 0.53 (*ns*) |
| Soil-N content (g $kg^{-1}$) | 8.1 | 1.1 | 0.34 (*ns*) | 0.54 (*ns*) | 8.3 | 1.0 | 0.42 (*ns*) | 0.53 (*ns*) |
| Soil-C content (g $kg^{-1}$) | 3.2 | 0.4 | 0.79 (*ns*) | 0.79 (*ns*) | 6.6 | 0.8 | 0.54 (*ns*) | 0.54 (*ns*) |
| Soil-Mg content [cmol (+) $kg^{-1}$] | 12.2 | 2.0 | 0.04 (*) | 0.19 (*ns*) | 9.6 | 1.2 | 0.33 (*ns*) | 0.53 (*ns*) |
| Soil-Al content [cmol (+) $kg^{-1}$] | 3.5 | 0.5 | 0.75 (*ns*) | 0.79 (*ns*) | 10.8 | 1.3 | 0.26 (*ns*) | 0.53 (*ns*) |
| Soil-K content [cmol (+) $kg^{-1}$] | 14.3 | 2.1 | 0.04 (*) | 0.19 (*ns*) | 9.5 | 1.2 | 0.32 (*ns*) | 0.53 (*ns*) |
| Soil-Ca content [cmol (+) $kg^{-1}$] | 7.2 | 1.2 | 0.29 (*ns*) | 0.54 (*ns*) | 11.0 | 1.2 | 0.25 (*ns*) | 0.53 (*ns*) |
| Soil-Pi content (mg $kg^{-1}$) | 4.5 | 0.8 | 0.64 (*ns*) | 0.79 (*ns*) | 11.3 | 1.3 | 0.25 (*ns*) | 0.53 (*ns*) |
| | Cropping | | | | | | | |
| Soil-pH | 5.6 | 0.8 | 0.57 (*ns*) | 0.86 (*ns*) | 13.3 | 1.8 | 0.18 (*ns*) | 0.48 (*ns*) |
| Soil-N content (g $kg^{-1}$) | 4.5 | 0.6 | 0.75 (*ns*) | 0.86 (*ns*) | 5.3 | 1.1 | 0.37 (ns) | 0.50 (*ns*) |
| Soil-C content (g $kg^{-1}$) | 10.4 | 1.5 | 0.17 (*ns*) | 0.46 (*ns*) | 19.9 | 2.5 | 0.01 (*) | 0.08 (*ns*) |
| Soil-Mg content [cmol (+) $kg^{-1}$] | 15.9 | 1.9 | 0.04 (*) | 0.17 (*ns*) | 8.8 | 1.2 | 0.32 (*ns*) | 0.50 (*ns*) |
| Soil-Al content [cmol (+) $kg^{-1}$] | 3.0 | 0.3 | 0.86 (*ns*) | 0.86 (*ns*) | 10.6 | 1.4 | 0.26 (*ns*) | 0.50 (*ns*) |
| Soil-K content [cmol (+) $kg^{-1}$] | 8.8 | 1.2 | 0.32 (*ns*) | 0.65 (*ns*) | 6.8 | 0.9 | 0.47 (*ns*) | 0.50 (*ns*) |
| Soil-Ca content [cmol (+) $kg^{-1}$] | 16.2 | 2.1 | 0.02 (*) | 0.17 (*ns*) | 3.7 | 0.4 | 0.47 (*ns*) | 0.53 (*ns*) |
| Soil-Pi content (mg $kg^{-1}$) | 3.9 | 0.5 | 0.86 (*ns*) | 0.86 (*ns*) | 17.6 | 3.6 | 0.02 (*) | 0.08 (*ns*) |

**Supplementary Table 3.** Effects of different individual soil chemical parameters as predictors on the arbuscular mycorrhizal community composition using the combined data from two sampling depths (0−10 cm and 10−20 cm) of different soil types (*Typic Kandiudox*, *Typic Kandiudult* and *Rhodic Kandiudult*) and fallow types (forest fallow, short fallow and cropping fields). Reported significances for *f*-values were determined based on the Monte Carlo permutation with 499 permutations test. Asterisks indicate statistical significance. *$p < 0.05$; **$p < 0.01$; ***$p < 0.001$; *ns*, not significant ($p \geq 0.05$). *p* (adjusted) values report the significance for multiple comparisons among the different soil chemical parameters analyzed on the basis of false discovery rate.

| | Variation explained (%) | *f*-value | *p*-value | *p*-adjusted |
|---|---|---|---|---|
| Soil-Ca content [cmol (+) $kg^{-1}$] | 8.3 | 6.3 | 0.002 (**) | 0.008 (**) |
| Soil-Mg content [cmol (+) $kg^{-1}$] | 6.3 | 4.7 | 0.006 (**) | 0.009 (**) |
| Soil-C content [g $kg^{-1}$] | 6.0 | 4.4 | 0.004 (**) | 0.008 (**) |
| Soil-pH | 5.6 | 4.1 | 0.004 (**) | 0.008 (**) |
| Soil-N content [g $kg^{-1}$] | 5.4 | 4.0 | 0.004 (**) | 0.008 (**) |
| Soil-K content [cmol (+) $kg^{-1}$] | 4.4 | 3.2 | 0.08 (*ns*) | 0.10 (*ns*) |
| Soil-Pi content [mg $kg^{-1}$] | 2.4 | 1.7 | 0.13 (*ns*) | 0.148 (*ns*) |
| Soil-Al content [cmol (+) $kg^{-1}$] | 1.2 | 0.9 | 0.48 (*ns*) | 0.474 (*ns*) |

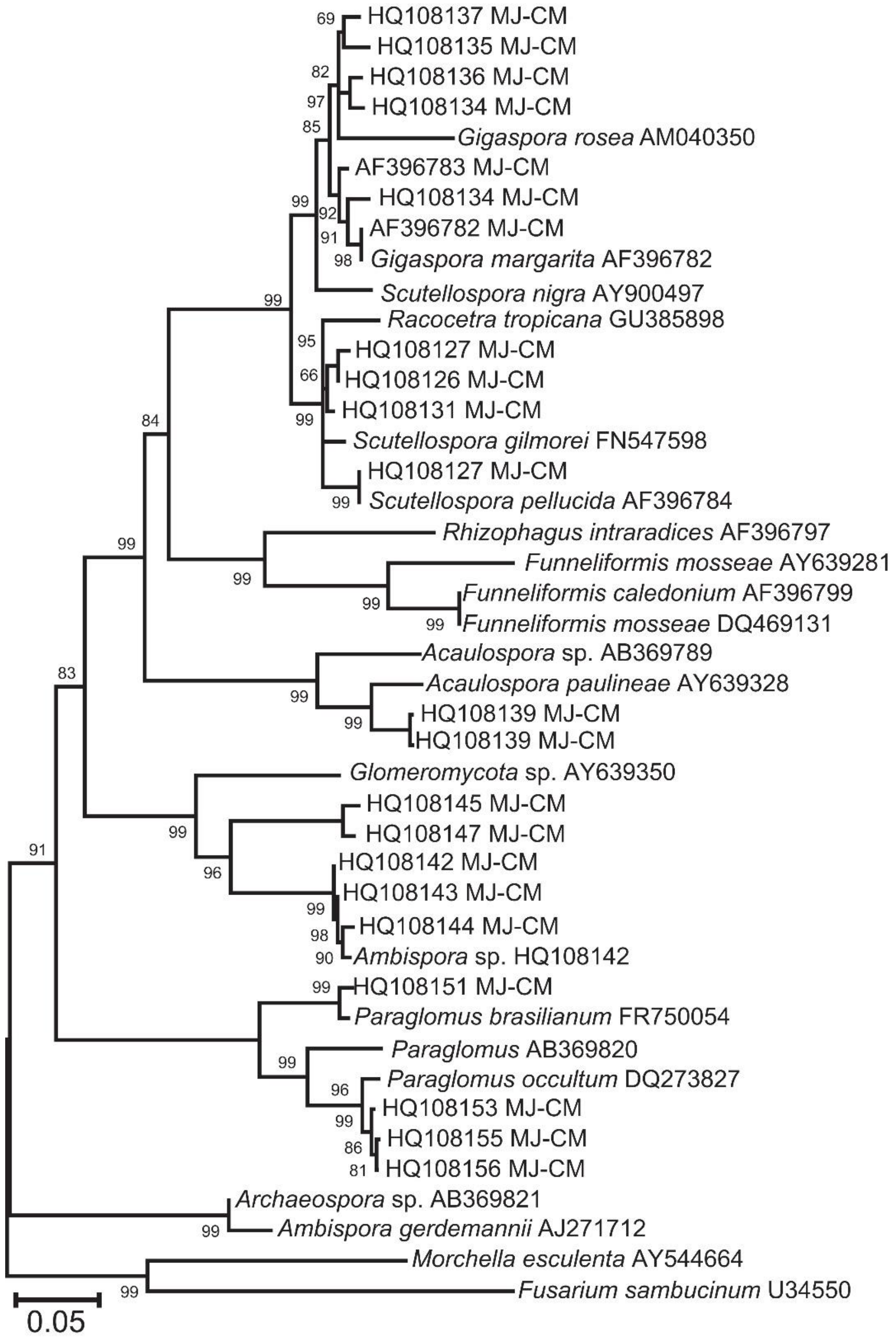


**Supplementary Figure 1.** Phylogenetic tree of the 28S rDNA sequences showing the relationship of the arbuscular mycorrhizal species isolated from two sampling depths (0–10 cm and 10–20 cm) of different soil types (*Typic Kandiudox*, *Typic Kandiudult* and *Rhodic Kandiudult*) and fallow types (forest fallow, short fallow and cropping field). Reference sequences are shown with full names and GenBank accession numbers. Newly obtained sequences are presented by their GenBank accession number. MJ-CM following each of the newly obtained sequences indicates the origin of sampled arbuscular mycorrhizal isolates. The bootstrap values indicate the support of the branches (percentage of 1000 permutations), and only the values above 60% are shown.

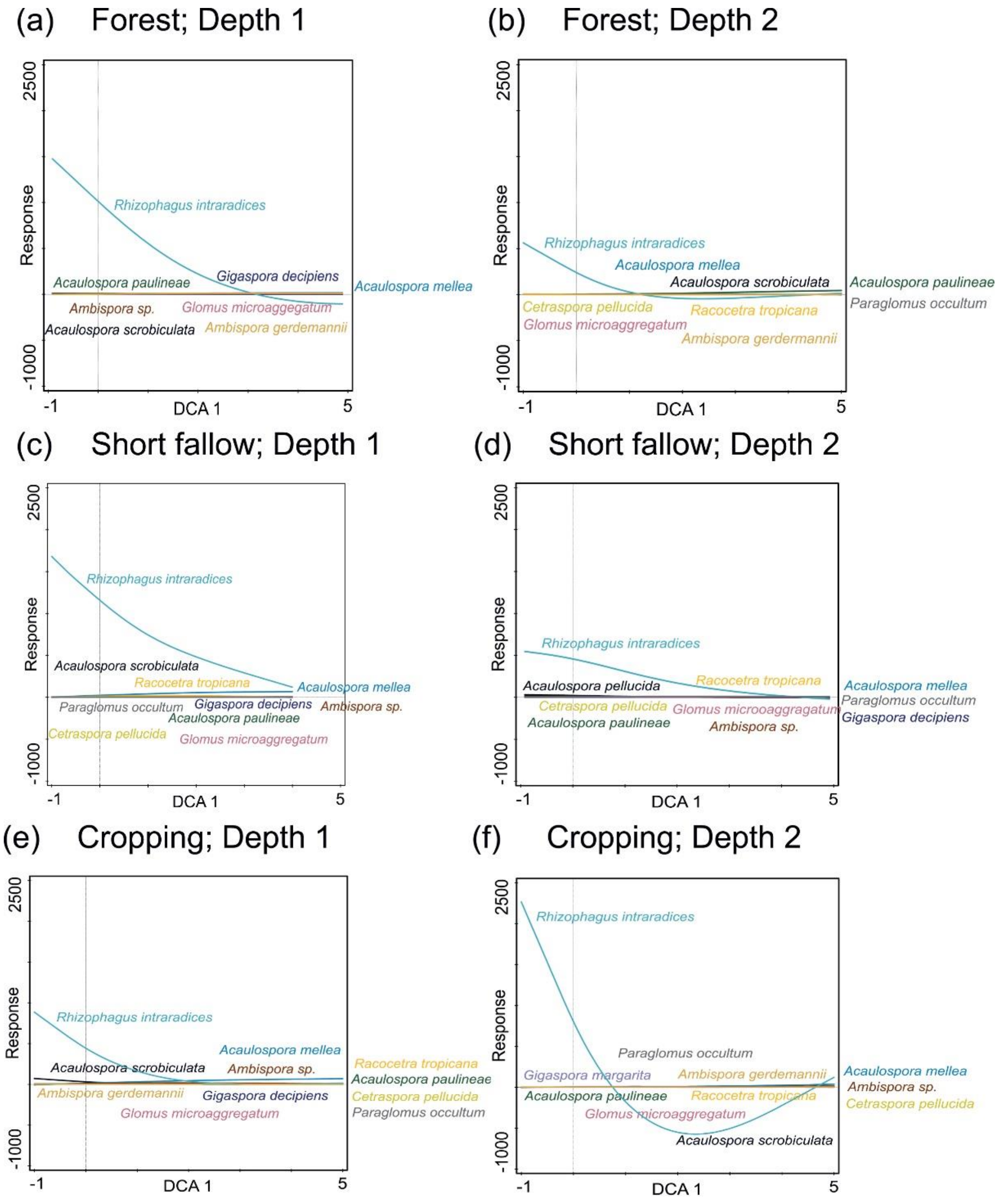


**Supplementary Figure 2.** Response curves of individual arbuscular mycorrhizal species fitted against the first detrended correspondence analysis (DCA1) axis by fallow types (forest fallow, short fallow and cropping fields) and sampling depths (0–10 cm and 10–20 cm). (a,b) Forest fallow, (c,d) short fallow, and (e,f) cropping fields with the two sampling depths (Depth 1, 0–10 cm; Depth 2, 10–20 cm).

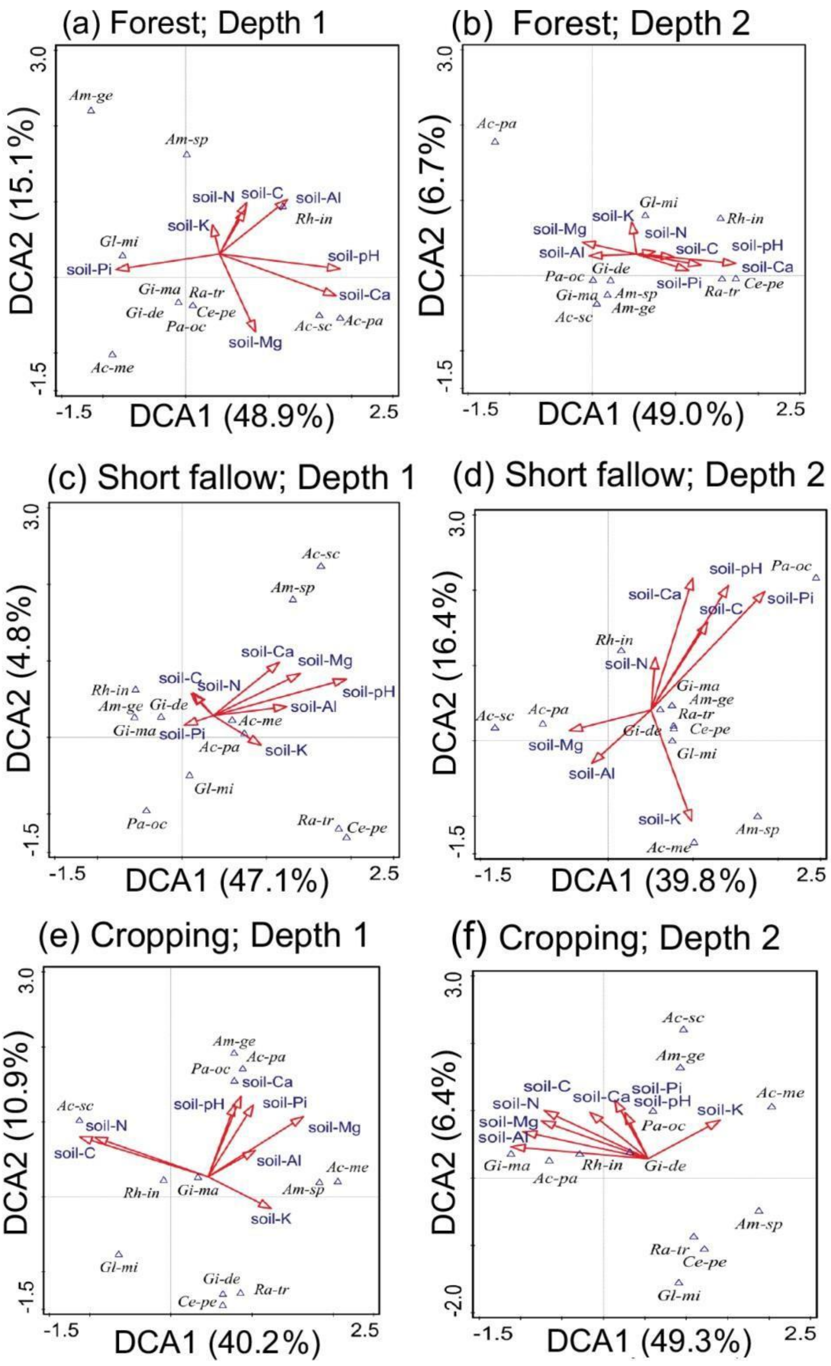


**Supplementary Figure 3.** Biplots of the detrended correspondence analysis (DCA) of the soil parameter variables on the arbuscular mycorrhizal community composition by fallow types (forest fallow, short fallow and cropping fields) and sampling depths (0-10 and 10-20 cm). (a,b) Forest fallow, (c,d) short fallow, and (e,f) cropping fields with the two sampling depths (Depth 1, 0-10 cm; Depth 2, 10-20 cm). Soil-pH, pH measured in soil suspension; soil- N, nitrogen content; soil-C, carbon content; soil-Mg, magnesium content; soil-Al, aluminium content; soil-K, potassium content; soil-Ca, calcium content; soil-Pi, inorganic phosphate content in soil; *Ac-sc*, *Acaulospora scrobiculata*; *Ac-me*, *Acaulospora mellea*; *Ac-pa*, *Acaulospora paulineae*; *Pa-oc*, *Paraglomus occultum*; *Ce-pe*, *Cetraspora pellucida*; *Ra-tr*, *Racocetra tropicana*; *Gi-de*, *Gigaspora decipiens*; *Gi-ma*, *Gigaspora margarita*; *Gl-mi*, *Glomus microaggregatum*; *Rh-in*, *Rhizophagus intraradices*; *Am-ge*, *Ambispora gerdemannii*; *Am*-sp, *Ambispora* sp..
